# Improved synthesis and application of conjugation-amenable polyols from d-mannose†

**DOI:** 10.1039/c9ra10378c

**Published:** 2020-01-23

**Authors:** Ida Mattsson, Ruzal Sitdikov, Andreas C. M. Gunell, Manu Lahtinen, Tiina Saloranta-Simell, Reko Leino

**Affiliations:** Laboratory of Molecular Science and Technology, Johan Gadolin Process Chemistry Centre, Åbo Akademi University FI-20500 Turku Finland reko.leino@abo.fi; Department of Chemistry, University of Jyväskylä FI-40014 Jyväskylä Finland

## Abstract

A series of polyhydroxyl sulfides and triazoles was prepared by reacting allyl and propargyl d-mannose derivatives with selected thiols and azides in thiol–ene and Huisgen click reactions. Conformational analysis by NMR spectroscopy proved that the intrinsic rigidity and linear conformation of the mannose derived polyol backbone is retained in the final click products in solution. Single crystal X-ray structure determination of one of the compounds prepared further verified that the linear conformation of the polyol segment is also retained in the solid state. In addition, an improved method for direct Barbier-type propargylation of unprotected d-mannose is reported. The new reaction protocol, involving tin-mediated propargylation in an acetonitrile-water mixture, provides access to multigram quantities of the desired, valuable alkyne polyol without relying on protecting group manipulations or chromatographic purification.

## Abstract 2

An improved method for the propargylation of d-mannose and application of the rod-like polyol and its allylated analogue in click reactions is described.

## Graphical abstract

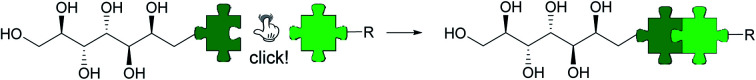

## Introduction

**Fig. 1:**
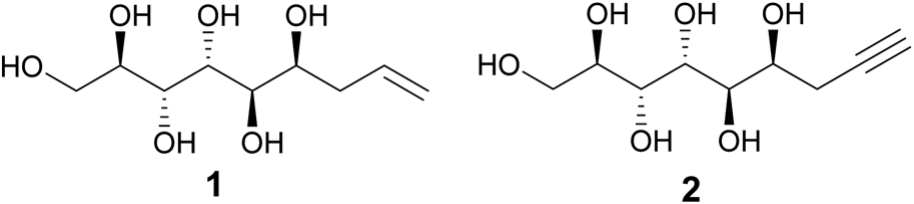
Chemical structures of allylated (1) and propargylated (2) d-mannose.

We have previously reported that the major diastereomer formed in the metal-mediated allylation of mannose (compound 1, Fig. 1) aggregates from aqueous solution upon stirring.^1,2^ This counter-intuitive, self-assembling behavior was shown to originate from the linear, planar zigzag conformation of the polyol backbone, retained both in the solid state and in solution, allowing the formation of highly ordered hydrogen bonded networks resulting in rod-like packing in the crystal lattice.^1,2^ Furthermore, both the propargylated analogue (compound 2, Fig. 1) and the hydrogenated congener of 1 exhibit similar behavior, likewise adopting linear solution and, as determined for compound 2, also solid state conformations.^2^ In contrast, as shown in the earlier study, the corresponding allylated d-glucose and d-galactose analogues adopted non-linear solution conformations without spontaneous aggregation, further manifesting the configurational uniqueness of the mannose derivative 1.^1^ The earlier conformational investigations also suggest that the linearity of the polyol backbone of these mannose-derived compounds is mainly responsible for the observed self-assembling and self-aggregating solution and solid-state behavior. Consequently, a relevant research question is whether the hydrophobic end of compounds 1 and 2 could be further derivatized, without affecting the intrinsic linear conformation and hydrogen-bonding properties of the polyol part of the molecule. This, in turn, would open the possibilities to utilize these mannose derived polyols as rigid and compact linear rods in various polymer science and material applications, tentatively, for example, in rod-coil block copolymers or liquid crystal systems.^3^

Click chemistry is a term coined by Sharpless in the early 2000s.^4^ Click reactions are characterized by high yields, insensitivity to water and oxygen, wide scope, regio- and stereo-specificity, simple workup and high atom economy. Among the most well known click-type reactions are the thiol–ene coupling and the Huisgen 1,3-dipolar cycloaddition reaction. Due to their inherent simplicity and versatility, these reactions have been extensively used in a wide range of applications, including medicinal chemistry, biochemistry and material sciences.^5^ En route to developing novel functional materials based on the mannose-derived, self-assembling linear rods, we report here our initial investigation on the utilization of the unsaturated functionalities of compounds 1 and 2 in click-reactions and the preparation and characterization of a series of their thiol and triazole derivatives. By NMR-spectroscopic conformational analysis, further supported by X-ray structure determination of one of the reaction products, we demonstrate here that, importantly, the linear conformation of the polyol backbone remains unaffected and stays intact in the final products, both in solution and in solid state. In addition, an improved and efficient, protective group free synthesis procedure for preparation of the propargylated mannose derivative 2 is described herein, potentially enabling its future use in preparative scale in different applications.

## Results and discussion

While the preparation of allylated carbohydrate derivatives through metal-mediated Barbier-type reaction was reported almost three decades ago,^6^ the corresponding propargylation is less straightforward. Metal-mediated propargylation in general typically results in mixtures of the propargylic and allenic products due to facile rearrangement of the triple bond.^7^ While different reaction protocols for selective propargylation of carbonyl compounds have been developed previously,^7^ to our knowledge, successful methods for direct metal-mediated propargylation of C1 in unmodified monosaccharides have not been described. For propargylation of d-mannose, all earlier reported methods are suboptimal, involving either complex isolation procedures, multiple steps and protecting groups, or expensive chiral ligands.^2,8^ For applying the Huisgen 1,3-cycloaddition click reaction to the propargylated mannose derivative in gram scale, an efficient synthesis procedure becomes essential.

**Scheme 1:**
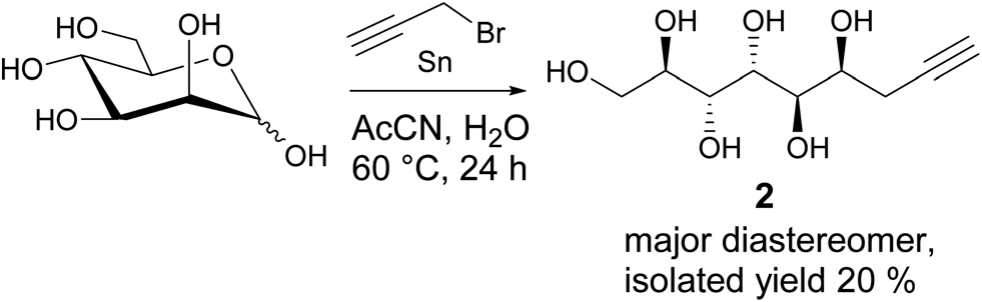
Metal-mediated propargylation of d-mannose.

For optimization of our earlier reported method,^2^ various reaction conditions (solvent, metal, temperature, reaction time, optimization is shown in Table S1 in ESI†) for Barbier-type, metal-mediated propargylation of d-mannose were screened. It was found that conducting the reaction with fresh reagents in degassed AcCN : H_2_O 9 : 1, under argon atmosphere, produces the diastereomerically pure propargylated compound 2 in 20% isolated yield (Scheme 1). The seemingly low yield is, however, well compensated by the simplicity of the synthesis protocol which allows to isolate the desired compound 2 in pure form, in multigram scale, by simple crystallization from water, and without any protecting group manipulations or chromatographic purification. Unfortunately, even at longer reaction times, complete conversion of the starting material is not achieved with the NMR spectra of the crude reaction product showing a complex mixture of multiple products and possible degradation. Also a small amount of the allenic analogue forms during the reaction and co-precipitates with the desired propargylated product (see peaks at approximately 5.4, 4.9 and 4.5 ppm in the ^1^H-NMR spectrum in ESI†). This is, however, not a critical issue for further use of the propargylic product in copper-catalyzed Huisgen 1,3-cycloaddition reaction, as the allene derivative is inactive in such reactions and can be removed at the work-up stage by simple washing of the triazol products with water.

**Fig. 2:**
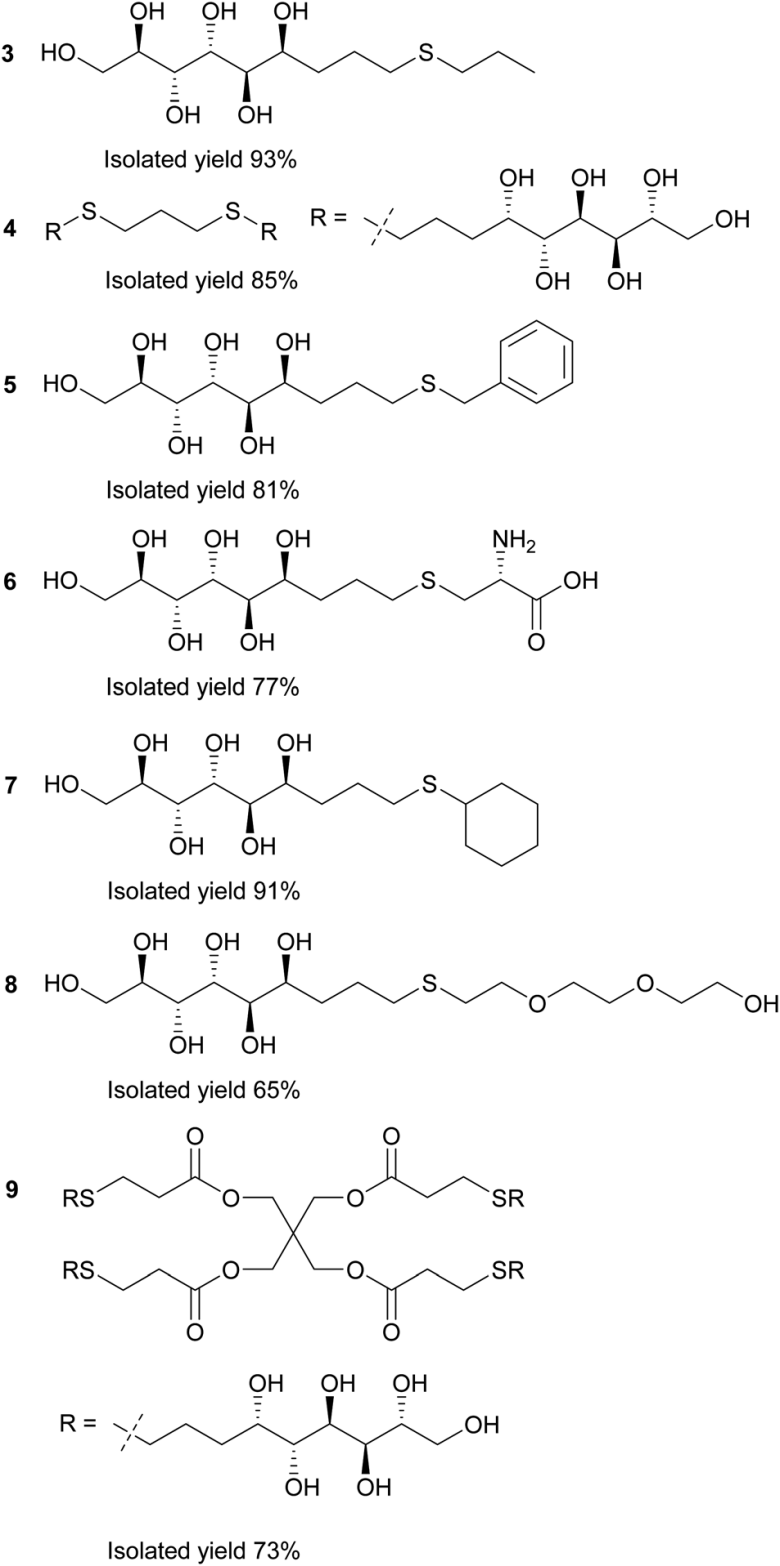
Reaction products of the UV-induced thiol–ene click reactions between thiols and allylated d-mannose (1).

**Scheme 2:**
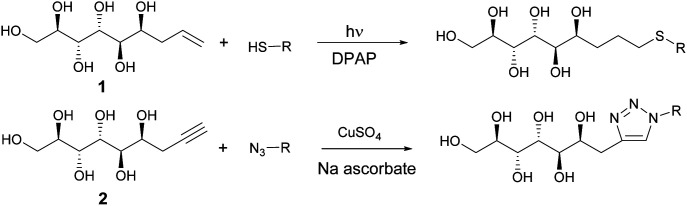
Synthesis of sulphides and triazoles from mannose derivatives by thiol–ene and Huisgen click reactions.

In the first stage of the click-reaction screening, sulfides 3–9 (Fig. 2) were prepared in good to excellent isolated yields *via* the UV-initiated thiol–ene reaction (Scheme 2). The reactions were conducted by irradiating solutions of allylated d-mannose derivative 1 and the corresponding thiols in H_2_O/MeOH or H_2_O/DMF mixtures with 365 nm 125 W UV-light, using 2,2-dimethoxy-2-phenylacetophenone (DPAP) as the initiator. Full conversions were typically obtained within 60 minutes. The work-up procedure was notably easy: the products were purified by washing the solid crude products with suitable solvents in order to remove excess starting materials and initiator residuals, followed by centrifugation/decantation and freeze-drying. For full experimental procedures and spectral data, see Experimental section and ESI.†

**Fig. 3:**
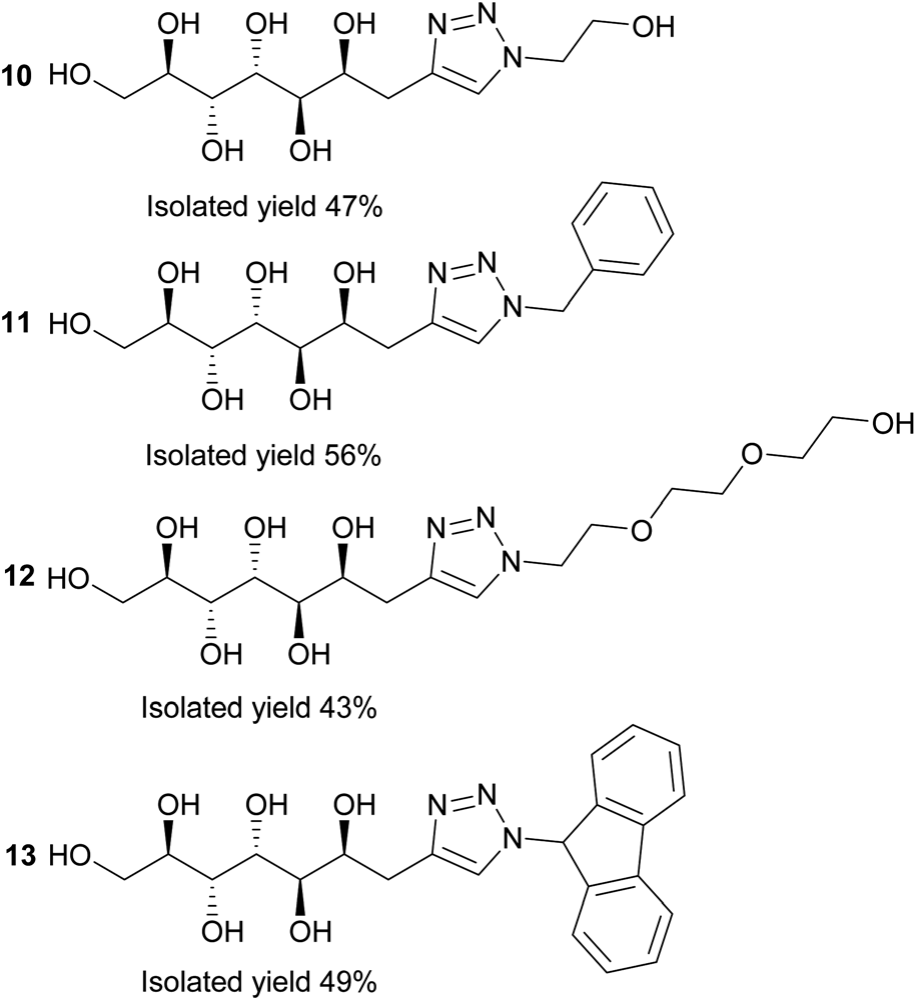
Reaction products of the copper-catalyzed Huisgen 1,3-cycloaddition click reactions between azides and propargylated d-mannose (2).

Next, the propargylated mannose derivative 2 was coupled to a series of azides *via* the copper-catalyzed Huisgen 1,3-cycloaddition reaction (Scheme 2) to form the triazoles 10–13 (Fig. 3). The coupling reactions were carried out in H_2_O, H_2_O/THF or DMF/H_2_O mixtures at 55 °C. The work-up was conducted in a similar manner as for the sulfides, by washing with suitable organic solvent and water, followed by centrifugation/decantation and freeze-drying. Although full conversions were typically reached, the isolated yields were only moderate due to the relatively high solubility of the products in water, causing partial dissolution of the reaction products and material losses upon removing the salts formed. For full experimental details, see Experimental section and ESI.†

To reassure that no changes in the conformation of the polyol backbone occurs upon chain elongation, all prepared compounds underwent conformational analysis by NMR spectroscopy. The relationship between the size of the vicinal ^3^*J*_H,H_ couplings and the conformation of acyclic polyols has been thoroughly elucidated in previous literature.^9^ For polyol compounds in linear conformation, the ^3^*J*_H,H_ couplings should be either small (<3 Hz), corresponding to a dihedral angle of 60°, or large (7–10 Hz), corresponding to a dihedral angle of 180°, consistent with linearity. Intermediate ^3^*J*_H,H_ couplings (3–7 Hz) instead indicate that the carbon chain is twisted rather than linear. All prepared compounds were fully characterized by NMR spectroscopy, and the ^1^H–^1^H coupling constant patterns were compared with the corresponding data for the original polyols 1 and 2.^1,2^ In our earlier studies, it was shown that the vicinal coupling constants for linear mannose-based polyols follow a regular pattern, being either small (≤1.6 Hz) when the neighboring protons are in *gauche* configuration, or large (8.3–9.5 Hz) when the neighboring protons are in *anti* configuration.^1,2^ The sulfides and triazoles prepared in this work did not exhibit any significant changes in the coupling constant pattern of the polyol backbone (see Table 1), consistent with the linear conformation of the polyol part indeed being retained also with longer, flexible chains attached to the molecules. Similar to the starting polyols, the linearity of the sulfides and triazoles does not appear to be solvent-dependent, being retained both in D_2_O and in d_6_-DMSO.^2^

**Table 1:** Vicinal coupling constants (Hz) for the polyol backbone of the mannose-derivatives

Compound	*J*_2,3_	*J*_3,4_	*J*_4,5_	*J*_5,6_
1^a^	8.9	1.1	9.4	1.5
1^b^	8.3	<1	9.3	1.6
2^a^	8.9	1.1	9.5	1.5
2^b^	8.3	1.0	9.3	1.5
3^a^	8.8	<1	9.4	1.4
4^b^	8.2	<1	9.3	1.4
5^b^	8.0	<1	9.2	1.2
6^a^	8.9	1.1	9.3	1.5
7^b^	8.0	<1	9.3	1.3
8^a^	8.8	<1	9.3	1.1
9^b^	8.4	<1	9.1	1.4
10^b^	8.7	<1	8.9	1.3
11^b^	8.4	<1	8.8	1.5
12^b^	8.9	<1	8.8	1.4
13^b^	8.8	1.4	9.5	<1

a Coupling constants in D_2_O.

b Coupling constants in d_6_-DMSO. Complete NMR-spectroscopic data for compounds 1 and 2 are found in ref. 1 and 2.

**Fig. 4:**
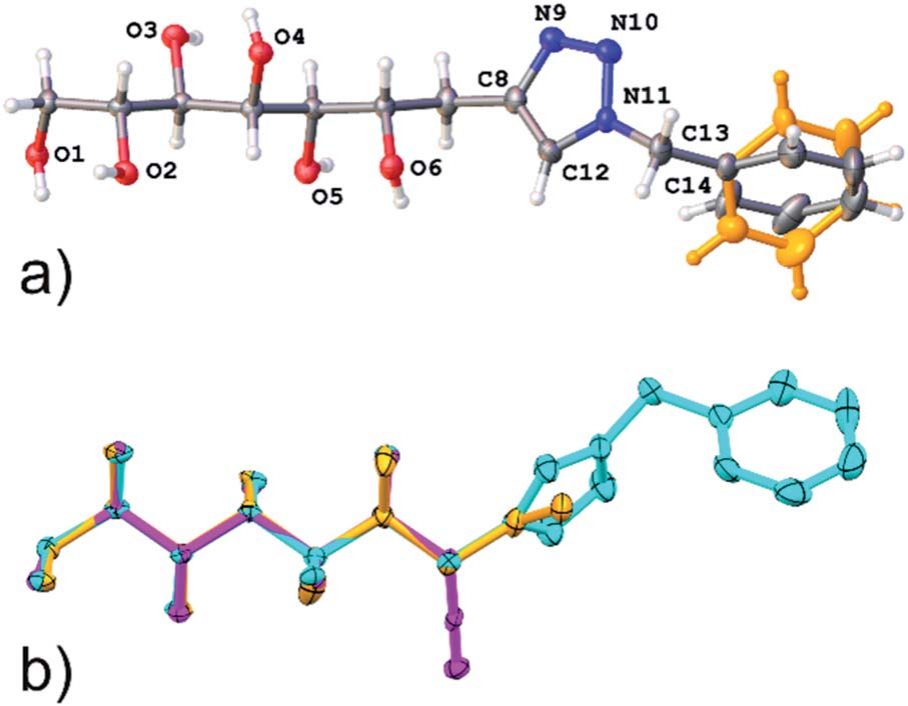
(a) Illustration of an asymmetric unit of crystal structure of compound 11, disordering of phenyl group is highlighted in orange color; (b) overlay of the structures of compound 11 and the previously reported allylated (orange)- and propargylated (magenta) d-mannoses.^1,2^

In addition, the single crystal X-ray structure of compound 11 was determined. Compound 11 crystallizes in a triclinic crystal system in chiral space group *P*1, with one molecule in the asymmetric unit (Fig. 4). In solid state, the carbohydrate backbone of 11 adopts a linear conformation similar to the previously reported structures of the allylated (1) and propargylated (2) derivatives of d-mannose,^1,2^ as can be observed from the well matching molecular overlap of all three compounds shown in Fig. 4. The intermolecular packing of the molecules in the crystal lattice is set by O–H⋯O, O–H⋯N and C–H⋯N hydrogen bonding (see Table S3, ESI†), thereby arranging the individual molecules in the same way, side by side along the *a*-and *b*-axes resulting in head to tail order along the *c*-axis. O–H⋯O hydrogen [*d*(D–A) = ∼2.7 Å] bonding occurs between the hydroxyl groups (O1, O2, O3, O4 and O5) of adjacent carbohydrate backbone hydroxyl groups. The C–H⋯N hydrogen bond is manifested between two adjacent triazole groups (C12–H12⋯N10) and O–H⋯N between a hydroxyl (O6) group and a triazole ring nitrogen (N9). The molecular packing and hydrogen bond network are illustrated in Fig. S2, ESI.† It can also be observed that the phenyl group is disordered over to equally occupied sites (shown in orange in Fig. 4). This is manifested in the packing scheme as an orientation of two adjacent phenyl groups in a manner of weak phase-to-edge π–π interaction. Although the actual distances between the phenyl ring centroids (∼4.8–5.3 Å, *vs.* mainly <5.5 Å) are within the generally accepted π–π interaction distance, the somewhat too low contact angle (∼40° *vs.* mainly >60°) between the ring planes indicates weak interaction.

## Experimental

### Instrumentation and chemicals

All NMR spectra were recorded at 298 K on a Bruker Avance-III HD 500 MHz spectrometer equipped with a Bruker SmartProbe™. Deuterated DMSO with 0.03% tetramethylsilane (TMS) as internal standard or D_2_O were used as solvents. Coupling constants were solved with Chemadder/SpinAdder software, The Spin Discoveries Ltd.^10^ HRMS were recorded with a Bruker Daltonics micro-QToF instrument in positive or negative mode using ESI-ionization. All UV-induced reactions were conducted in a 75 ml immersion well reactor equipped with a 125 W lamp emitting UV light at 365 nm wavelength. The reactor system was purchased from Photochemical Reactors LTD. The reactor setup is shown in Fig. S1, ESI.† All chemicals and solvents were purchased from commercial vendors and were used as such without purification.

### Synthesis of (2*R*,3*R*,4*R*,5*R*,6*S*)-non-8-ene-1,2,3,4,5,6-hexaol 1

Compound 1 was prepared according to previously published protocols.^1,2,6^ For full details, see ESI.†

### Synthesis of (2*R*,3*R*,4*R*,5*R*,6*S*)-non-8-yne-1,2,3,4,5,6-hexaol 2

d-Mannose (5 g, 27.8 mmol, 1 eq.) was dissolved in a mixture of degassed AcCN (550 ml) and distilled H_2_O (50 ml) under argon atmosphere. Tin powder (6.7 g, 56.4 mmol, 2 eq.) and propargyl bromide 80% in toluene solution (9.3 ml, 83.3 mmol, 3 eq.) were added and the reaction mixture was heated to 60 °C and stirred overnight. The reaction mixture was allowed to cool down to room temperature, and was subsequently filtered through Celite. The desired product precipitates during the reaction, allowing to discard the brown-colored AcCN filtrate. The filter cake was then washed with 1 l boiling water. The aqueous filtrate was concentrated *in vacuo* until about 80 ml of the liquids remained. The residue was placed in a refrigerator (+4 °C) overnight allowing the compound 2 (1.2 g, 5.45 mmol) to precipitate as white crystals, corresponding to an isolated yield of 20%. As the propargylation reaction appears significantly more air sensitive than the corresponding allylation, freshly prepared reagents should be used. ^1^H-NMR (500.20 MHz, D_2_O, 25 °C): *δ* = 4.12 (ddd, *J*_6,5_ = 1.5 Hz, *J*_6,7a_ = 7.6 Hz, *J*_6,7b_ = 6.6 Hz, 1H, H-6), 3.91 (dd, *J*_4,3_ = 1.1 Hz, *J*_4,5_ = 9.5 Hz, 1H, H-4), 3.88 (dd, *J*_1a,1b_ = −11.9 Hz, *J*_1a,2_ = 2.9 Hz, 1H, H-1a), 3.83 (dd, *J*_3,2_ = 8.9 Hz, 1H, H-3), 3.78 (ddd, *J*_2,1b_ = 6.5 Hz, 1H, H-2), 3.73 (dd, 1H, H-5), 3.68 (dd, 1H, H-1b), 2.56 (ddd, *J*_7a,7b_ = −16.8 Hz, *J*_7a,9_ = 2.6 Hz, 1H, H-7a), 2.51 (ddd, *J*_7b,9_ = 2.7 Hz, 1H, H-7b), 2.40 (dd, 1H, H-9) ppm. ^13^C-NMR (125.8 MHz, D_2_O, 25 °C): *δ* = 82.0 (C-8), 70.9 (C-9), 70.8 (C-2), 70.4 (C-5), 69.3 (C-3), 68.5 (C-6), 68.4 (C-4), 63.3 (C-1), 23.0 (C-7) ppm. HRMS calcd. for C_9_H_16_O_6_Na [M + Na]^+^: 243.0839, found: 243.0873.

### General procedure for preparation of compounds 3–9

Allylated d-mannose (1) (100 mg, 0.45 mmol, 1 eq.), thiol (0.9–2 eq.) and 2,2-dimethoxy-2-phenylacetophenone (5.8 mg, 0.023 mmol, 0.05 eq.) were dissolved in 10 ml MeOH : H_2_O 1 : 1. The reaction mixture was irradiated with 365 nm UV-light for 1 h. Next, the mixture was evaporated to dryness and the solids were stirred in acetone, ethyl acetate, toluene or hexane solution for 1 h in order to remove residual thiol and initiator. The mixture was then centrifuged, decanted and dried *in vacuo*. The products were isolated as off-white/white powders. Full experimental details are found in ESI.†

#### (2*R*,3*R*,4*R*,5*R*,6*S*)-9-(Propylthio)nonane-1,2,3,4,5,6-hexaol (3)

White powder, 93%.^1^H-NMR (500.20 MHz, D_2_O, 25 °C): *δ* = 3.89 (m, 1H, H-6), 3.88 (dd, *J*_4,5_ = 9.4 Hz, *J*_4,3_ = <1 Hz, 1H, H-4), 3.85 (dd, *J*_1b,2_ = 2.9 Hz, *J*_1a,1b_ = −11.9 Hz, 1H, H1a), 3.79 (dd, *J*_3,2_ = 8.8 Hz, 1H, H-3), 3.74 (ddd, *J*_2,1b_ = 6.4 Hz, 1H, H-2), 3.65 (dd, 1H, H-1b), 3.53 (dd, *J*_5,6_ = 1.4 Hz, 1H, H-5), 2.60 (m, 2H, H-9), 2.55 (t, *J*_10,11_ = 7.2 Hz, 2H, H-10), 1.70 (m, 1H, H-8a), 1.67 (m, 1H, H-7a), 1.66 (m, 1H, H-8b), 1.61 (m, 1H, H-7b), 1.58 (m, 2H, H-11), 0.93 (t, *J*_12,11_ = 7.4 Hz, 3H, H-12) ppm. ^13^C-NMR (125.8 MHz, D_2_O, 25 °C): *δ* = 71.5 (C-5), 71.0 (C-2), 69.42 (C-6), 69.37 (C-3), 68.5 (C-4), 63.3 (C-1), 33.2 (C-10), 32.1 (C-7), 30.9 (C-9), 25.4 (C-8), 22.3 (C-11), 12.7 (C-12) ppm. HRMS calcd. for C_12_H_25_O_6_S [M − H]^−^: 297.1366, found: 297.1397.

#### (2*R*,2′*R*,3*R*,3′*R*,4*R*,4′*R*,5*R*,5′*R*,6*S*,6′*S*)-9,9′-(Propane-1,3-diylbis(sulfanediyl))bis(nonane-1,2,3,4,5,6-hexaol) (4)

Off-white powder, 85%. ^1^H-NMR (500.20 MHz, DMSO, 25 °C): *δ* = 4.38 (d, *J*_OH2,2_ = 5.6 Hz, 2H, OH-2, OH-2′), 4.32 (t, *J*_OH1,1_ = 5.7 Hz, 2H, OH-1, OH-1′), 4.07 (d, *J*_OH5,5_ = 7.4 Hz, 2H, OH-5, OH-5′), 4.06 (d, *J*_OH4,4_ = 7.7 Hz, 2H, OH-4, OH-4′), 4.04 (d, *J*_OH3,3_ = 7.5 Hz, 2H, OH-3, OH-3′), 4.00 (d, *J*_OH6,6_ = 7.4 Hz, 2H, OH-6, OH-6′), 3.68 (ddd, *J*_4,3_ < 1 Hz, *J*_4,5_ = 9.3 Hz, 2H, H-4, H-4′), 3.67 (m, 2H, H-6, H-6′), 3.61 (m, 2H, H-1a, H-1a′), 3.56 (ddd, *J*_3,2_ = 8.2 Hz, 2H, H-3, H-3′), 3.47 (m, 2H, H-2, H-2′), 3.38 (m, 2H, H-1b, H-1b′), 3.25 (ddd, *J*_5,6_ = 1.4 Hz, 2H, H-5, H-5′), 2.57 (m, 4H, H-10, H-10′), 2.50 (m, under DMSO signal, 4H, H-9, H-9′), 1.76 (m, 2H, H-11), 1.65 (m, 2H, H-8a, H-8a′), 1.54 (m, 2H, H-8b, H-8b′), 1.54 (m, 2H, H-7a, H-7a′), 1.47 (m, 2H, H-7b, H-7b′) ppm. ^13^C-NMR (125.8 MHz, DMSO, 25 °C): *δ* = 71.4 (2 × C, C-2, C-2′), 71.3 (2 × C, C-5, C-5′), 69.6 (2 × C, C-3, C-3′), 68.9 (2 × C, C-6, C-6′), 68.7 (2 × C, C-4, C-4′), 63.8 (2 × C, C-1, C-1′), 32.8 (2 × C, C-7, C-7′), 31.2 (2 × C, C-9, C-9′), 29.9 (2 × C, C-10, C-10′), 29.0 (C-11), 26.0 (2 × C, C-8, C-8′) ppm. HRMS calcd. for C_21_H_43_O_12_S_2_ [M − H]^−^: 551.2190, found: 551.2195.

#### (2*R*,3*R*,4*R*,5*R*,6*S*)-9-(Benzylthio)nonane-1,2,3,4,5,6-hexaol (5)

Off-white powder, 81%. ^1^H-NMR (500.20 MHz, DMSO, 25 °C): *δ* = 7.2–7.4 (m, 5H, H-12, H12′, H-13, H-13′, H-14), 4.39 (d, *J*_OH2,2_ = 5.4 Hz, 1H, OH-2), 4.33 (t, *J*_OH1,1_ = 5.4 Hz, 1H, OH-1), 4.08 (d, *J*_OH4,4_ = 7.1 Hz, 1H, OH-4), 4.07 (d, *J*_OH5,5_ = 7.9 Hz, 1H, OH-5), 4.04 (d, *J*_OH3,3_ = 7.6 Hz, 1H, OH-3), 3.98 (d, *J*_OH6,6_ = 7.3 Hz, 1H, OH-6), 3.71 (s, 2H, H-10), 3.69 (ddd, *J*_4,3_ < 1 Hz, *J*_4,5_ = 9.2 Hz, 1H, H-4), 3.66 (m, 1H, H-6), 3.62 (m, 1H, H-1a), 3.56 (ddd, *J*_3,2_ = 8.0 Hz, 1H, H-3), 3.47 (m, 1H, H-2), 3.38 (m, 1H, H-1b), 3.25 (ddd, *J*_5,6_ = 1.2 Hz, 1H, H-5), 2.40 (m, 2H, H-9), 1.65 (m, 1H, H-8a), 1.54 (m, 1H, H-8b), 1.53 (m, 1H, H-7a), 1.45 (m, 1H, H7-b) ppm. ^13^C-NMR (125.8 MHz, DMSO, 25 °C): *δ* = 130.1 (C-11), 128.7 (2 × C, C-12, C12′), 128.2 (2 × C, C-13, C-13′), 126.6 (C-14), 71.4 (C-2), 71.3 (C-5), 69.6 (C-3), 68.9 (C-6), 68.6 (C-4), 63.9 (C-1), 34.9 (C-10), 32.8 (C-7), 30.8 (C-9), 25.5 (C-8) ppm. HRMS calcd. for C_16_H_26_O_6_SNa [M + Na]^+^: 369.1342, found: 369.1368.

#### *S*-((4*S*,5*R*,6*R*,7*R*,8*R*)-4,5,6,7,8,9-Hexahydroxynonyl)-l-cysteine (6)

White powder, 77%. ^1^H-NMR (500.20 MHz, D_2_O, 25 °C): *δ* = 3.93 (dd, *J*_11,10a_ = 4.3 Hz, *J*_11,10b_ = 7.5 Hz, 1H, H-11), 3.92 (m, 1H, H-6), 3.91 (dd, *J*_4,3_ = 1.1 Hz, *J*_4,5_ = 9.3 Hz, 1H, H-4), 3.87 (dd, *J*_1a,1b_ = −11.9 Hz, *J*_1a,2_ = 2.6 Hz, 1H, H-1a), 3.82 (dd, *J*_3,2_ = 8.9 Hz, 1H, H-3), 3.77 (ddd, *J*_2,1b_ = 6.3 Hz, 1H, H-2), 3.67 (dd, 1H, H-1b), 3.56 (dd, *J*_5,6_ = 1.5 Hz, 1H, H-5), 3.15 (dd, *J*_10a,10b_ = −14.8 Hz 1H, H-10a), 3.04 (dd, 1H, H-10b), 2.67 (dd, *J*_9,8_ = 6.5 Hz, *J*_9,8_ = 7.0 Hz, 2H, H-9), 1.73 (m, 2H, H-8), 1.72 (m, 1H, H-7a), 1.64 (m, 1H, H7b) ppm. ^13^C-NMR (125.8 MHz, D_2_O, 25 °C): *δ* = 173.0 (C-12), 71.5 (C-5), 71.0 (C-2), 69.37 (C-6), 69.35 (C-3), 68.5 (C-4), 63.3 (C-1), 53.6 (C-11), 31.92 (C-7), 31.91 (C-10), 31.2 (C-9), 25.2 (C-8) ppm. HRMS calcd. for C_12_H_24_NO_8_S [M − H]^−^: 342.1217, found: 342.1220.

#### (2*R*,3*R*,4*R*,5*R*,6*S*)-9-(Cyclohexylthio)nonane-1,2,3,4,5,6-hexaol (7)

Off-white powder, 91%. ^1^H-NMR (500.20 MHz, DMSO, 25 °C): *δ* = 4.38 (d, *J*_OH2,2_ = 5.5 Hz, 1H, OH-2), 4.32 (t, *J*_OH1,1_ = 5.7 Hz, 1H, OH-1), 4.08 (d, *J*_OH4,4_ = 7.2 Hz, 1H, OH-4), 4.06 (d, *J*_OH5,5_ = 7.9 Hz, 1H, OH-5), 4.04 (d, *J*_OH3,3_ = 7.5 Hz, 1H, OH-3), 3.98 (d, *J*_OH6,6_ = 7.4 Hz, 1H, OH-6), 3.67 (ddd, *J*_4,3_ < 1 Hz, *J*_4,5_ = 9.3 Hz, 1H, H-4), 3.66 (m, 1H, H-6), 3.61 (m, 1H, H-1a), 3.56 (ddd, *J*_3,2_ = 8.0 Hz, 1H, H-3), 3.46 (m, 1H, H-2), 3.38 (m, 1H, H-1b), 3.25 (ddd, *J*_5,6_ = 1.3 Hz, 1H, H-5), 2.65 (1H, H-10), 2.50 (m, under DMSO signal, 2H, H-9), 1.89 (m, 2H, H-11a, H11a′), 1.69 (m, 2H, H-13), 1.62 (m, 1H, H-8a), 1.55 (m, 2H, H12a, H12a′), 1.54 (m, 1H, H-8b), 1.53 (m, 1H, H-7a), 1.46 (m, 1H, H-7b), 1.25 (m, 2H, H-12b, H-12b′), 1.24 (m, 2H, H-11b, H11b′) ppm. ^13^C-NMR (125.8 MHz, DMSO, 25 °C): *δ* = 71.4 (C-2), 71.3 (C-5), 69.6 (C-3), 68.9 (C-6), 68.7 (C-4), 63.8 (C-1), 42.4 (C-10), 33.2 (C-11), 32.9 (C-7), 29.4 (C-9), 26.5 (C-8), 25.4 (C-13), 25.3 (C-12) ppm. HRMS calcd. for C_15_H_30_O_6_SNa [M + Na]^+^: 361.1655, found: 361.1646.

#### (2*R*,3*R*,4*R*,5*R*,6*S*)-9-((2-(2-(2-Hydroxyethoxy)ethoxy)ethyl)thio)nonane-1,2,3,4,5,6-hexaol (8)

Off-white powder, 65%. ^1^H-NMR (500.20 MHz, D_2_O, 25 °C): *δ* = 3.92 (m, 1H, H-6), 3.91 (dd, *J*_4,3_ < 1 Hz, *J*_4,5_ = 9.3 Hz, 1H, H-4), 3.87 (dd, *J*_1a,1b_ = −11.9 Hz, *J*_1a,2_ = 2.8 Hz, 1H, H-1a), 3.82 (dd, *J*_3,2_ = 8.8 Hz, 1H, H-3), 3.77 (ddd, *J*_2,1b_ = 6.3 Hz, 1H, H-2), 3.76–3.63 (m, 10H, H-11-H-15), 3.67 (dd, 1H, H-1b), 3.56 (dd, *J*_5,6_ = 1.1 Hz, 1H, H-5), 2.80 (t, *J*_10,11_ = 6.3 Hz, 2H, H-10), 2.67 (m, 2H, H-9), 1.75 (m, 1H, H-8a), 1.71 (m, 1H, H-7a), 1.68 (m, 1H, H-8b), 1.65 (m, 1H, H7b) ppm. ^13^C-NMR (125.8 MHz, D_2_O, 25 °C): *δ* = 71.5 (C-5), 71.0 (C-2), 69.5 (C-6), 69.4 (C-3), 68.5 (C-4), 63.3 (C-1), 32.0 (C-7), 31.2 (C-9), 30.4 (C-10), 25.4 (C-8), C-11-C-15: 71.7, 69.5 3 × C, 60.4 ppm. HRMS calcd. for C_15_H_31_O_9_S [M − H]^−^: 387.1682, found: 387.1643.

#### 2,2-Bis(((3-(((4*S*,5*R*,6*R*,7*R*,8*R*)-4,5,6,7,8,9 hexahydroxynonyl)thio)propanoyl)oxy)methyl)propane-1,3-diylbis(3-(((4*S*,5*R*,6*R*,7*R*,8*R*)-4,5,6,7,8,9-hexahydroxynonyl)thio)propanoate) (9)

Off-white powder, 73%, ^1^H-NMR data (500.20 MHz, DMSO, 25 °C): *δ* = 4.39 (d, *J*_OH2,2_ = 5.5 Hz, 4H, OH-2, OH-2′, OH-2′′, OH-2′′′), 4.32 (t, *J*_OH1,1_ = 5.7 Hz, 4H, OH-1, OH-1′, OH-1′′, OH-1′′′), 4.12 (s, 8H, H-13, H-13′, H-13′′, H-13′′′), 4.09 (d, *J*_OH4,4_ = 7.6 Hz, 4H, OH-4, OH-4′, OH-4′′, OH-4′′′), 4.07 (d, *J*_OH5,5_ = 7.9 Hz, 4H, OH-5, OH-5′, OH-5′′, OH-5′′′), 4.04 (d, *J*_OH3,3_ = 7.7 Hz, 4H, OH-3, OH-3′, OH-3′′, OH-3′′′), 3.99 (d, *J*_OH6,6_ = 7.6 Hz, 4H, OH-6, OH-6′, OH-6′′, OH-6′′′), 3.68 (ddd, *J*_4,3_ < 1 Hz, *J*_4,5_ = 9.1 Hz, 4H, H-4, H-4′, H-4′′, H-4′′′), 3.67 (m, 4H, H-6, H-6′, H-6′′, H-6′′′), 3.61 (m, 4H, H-1a, H-1a′, H-1a′′, H-1a′′′), 3.56 (ddd, *J*_3,2_ = 8.4 Hz, 4H, H-3, H-3′, H-3′′, H-3′′′), 3.47 (m, 4H, H-2, H-2′, H-2′′, H-2′′′), 3.38 (m, 4H, H-1b, H-1b′, H-1b′′, H-1b′′′), 3.25 (ddd, *J*_5,6_ = 1.4 Hz, 4H, H-5, H-5′, H-5′′, H-5′′′), 2.69 (m, 8H, H-10, H-10′, H-10′′, H-10′′′), 2.62 (m, 8H, H-11, H-11′, H-11′′, H-11′′′), 2.51 (m, under DMSO signal, 8H, H-9, H-9′, H-9′′, H-9′′′), 1.64 (m, 4H, H-8a, H-8a′, H-8a′′, H-8a′′′), 1.54 (m, 4H, H7-a, H7-a′, H7-a′′, H7-a′′′), 1.53 (m, 4H, H-8b, H-8b′, H-8b′′′′, H-8b′′′), 1.46 (m, 4H, H-7b, H-7b′, H-7b′′, H-7b′′′) ppm. ^13^C-NMR (125.8 MHz, DMSO, 25 °C): *δ* = 171.0 (4 × C, C-12, C-12′, C-12′′, C-12′′′), 71.4 (4 × C, C-2, C-2′, C-2′′, C-2′′′), 71.3 (4 × C, C-5, C-5′, C-5′′, C-5′′′), 69.6 (4 × C, C-3, C-3′, C-3′′, C-3′′′), 68.9 (4 × C, C-6, C-6′, C-6′′, C-6′′′), 68.7 (4 × C, C-4, C-4′, C-4′′, C-4′′′), 63.8 (4 × C, C-1, C-1′, C-1′′, C-1′′′), 62.0 (4 × C, C-13, C-13′, C-13′′, C-13′′′), 41.7 (C-14), 34.2 (4 × C, C-11, C-11′, C-11′′, C-11′′′), 32.8 (4 × C, C-7, C-7′, C-7′′, C-7′′′), 31.1 (4 × C, C-9, C-9′, C-9′′, C-9′′′), 26.0 (4 × C, C-8, C-8′, C-8′′, C-8′′′), 25.9 (4 × C, C-10, C-10′, C-10′′, C-10′′′) ppm. HRMS calcd. for C_53_H_100_O_32_S_4_ [M + Na]^+^: 1399.4972, found: 1399.5046.

### General procedure for synthesis of compounds 10–13

Propargylated d-mannose (2) (20 mg, 0.09 mmol, 1 eq.), azide (0.18 mmol, 2 eq.), copper(ii)sulfate (1.4 mg, 0.009, 0.1 eq.) and sodium ascorbate (3.6 mg, 0.018 mmol, 0.2 eq.) were dissolved in 5 ml H_2_O, THF : H_2_O or DMF : H_2_O. The solution was heated to 55 °C and stirred overnight. Next, the solvents were evaporated to dryness and the solids were washed with 2 ml toluene or ethyl acetate and 0.5 ml H_2_O to remove the residual azide and salts. The solids were separated from the liquid by centrifugation and decantation, followed by drying under reduced pressure. The products were obtained as white powders. Full experimental details are found in ESI.†

#### (2*R*,3*R*,4*R*,5*R*,6*S*)-7-(1-(2-Hydroxyethyl)-1*H*-1,2,3-triazol-4-yl)heptane-1,2,3,4,5,6-hexaol (10)

White powder, 47%. ^1^H-NMR (500.20 MHz, DMSO, 25 °C): *δ* = 7.79 (s, 1H, H-9), 5.05 (t, *J*_OH11,11_ = 5.3 Hz, 1H, OH-11), 4.42 (d, *J*_OH2,2_ = 5.5 Hz, 1H, OH-2), 4.35 (m, 2H, H-10), 4.34 (m, 1H, OH-1), 4.29 (d, *J*_OH5,5_ = 7.9 Hz, 1H, OH-5), 4.24 (d, *J*_OH6,6_ = 7.4 Hz, 1H, OH-6), 4.12 (d, *J*_OH4,4_ = 7.3 Hz, 1H, OH-4), 4.06 (d, *J*_OH3,3_ = 7.8 Hz, 1H, OH-3), 4.00 (dddd, *J*_6,5_ = 1.3 Hz, *J*_6,7_ = 6.9 Hz, 1H, H-6), 3.76 (m, 2H, H-11), 3.73 (ddd, *J*_4,3_ < 1 Hz, *J*_4,5_ = 8.9 Hz, 1H, H-4), 3.62 (m, 1H, H-1a), 3.57 (ddd, *J*_3,2_ = 8.7 Hz, 1H, H-3), 3.47 (m, 1H, H-2), 3.39 (m, 1H, H-1b), 3.33 (ddd, under H_2_O signal, 1H, H-5), 2.80 (d, 2H, H-7) ppm. ^13^C-NMR (125.8 MHz, DMSO, 25 °C): *δ* = 144.6 (C-8), 122.9 (C-9), 71.4 (C-2), 70.8 (C-5), 69.6 (C-3), 69.3 (C-6), 68.6 (C-4), 63.8 (C-1), 59.8 (C-11), 51.8 (C-10), 30.3 (C-7) ppm. HRMS calcd. for C_11_H_20_N_3_O_7_ [M − H]^−^: 306.1296, found: 306.1276.

#### (2*R*,3*R*,4*R*,5*R*,6*S*)-7-(1-Benzyl-1*H*-1,2,3-triazol-4-yl)heptane-1,2,3,4,5,6-hexaol (11)

Off-white powder, 56%. ^1^H-NMR (500.20 MHz, DMSO, 25 °C): *δ* = 7.85 (s, 1H, H-9), 7.37 (m, 2H, H-13, H-13′), 7.33 (m, 1H, H-14), 7.29 (m, 2H, H-12, H-12′), 5.55 (s, 2H, H-10), 4.39 (d, *J*_OH2,2_ = 5.5 Hz, 1H, OH-2), 4.33 (t, *J*_OH1,1_ = 5.7 Hz, 1H, OH-1) 4.25 (d, *J*_OH5,5_ = 7.7 Hz, 1H, OH-5), 4.20 (d, *J*_OH6,6_ = 7.7 Hz, 1H, OH-6), 4.09 (d, *J*_OH4,4_ = 7.3 Hz, 1H, OH-4), 4.01 (d, *J*_OH3,3_ = 7.8 Hz, 1H, OH-3), 3.97 (dddd, *J*_6,5_ = 1.5 Hz, *J*_6,7_ = 7.0 Hz, 1H, H-6), 3.72 (ddd, *J*_4,3_ < 1 Hz, *J*_4,5_ = 8.8 Hz, 1H, H-4), 3.61 (m, 1H, H-1a), 3.57 (ddd, *J*_3,2_ = 8.4 Hz, 1H, H-3), 3.46 (m, 1H, H-2), 3.38 (m, 1H, H-1b), 3.31 (ddd, under H_2_O signal, 1H, H-5), 2.79 (d, 2H, H-7) ppm. ^13^C-NMR (125.8 MHz, DMSO, 25 °C): *δ* = 145.3 (C-8), 136.3 (C-11), 128.7 (2 × C, C-13, C-13′), 128.0 (C-14), 127.8 (2 × C, C-12, C-12′), 122.8 (C-9), 71.5 (C-2), 70.9 (C-5), 69.7 (C-3), 69.4 (C-6), 68.7 (C-4), 63.9 (C-1), 52.6 (C-10), 30.5 (C-7) ppm. HRMS calcd. for C_16_H_23_N_3_O_6_Na [M + Na]^+^: 376.1479, found: 376.1482.

#### (2*R*,3*R*,4*R*,5*R*,6*S*)-7-(1-(2-(2-(2-Hydroxyethoxy)ethoxy)ethyl)-1*H*-1,2,3-triazol-4-yl)heptane-1,2,3,4,5,6-hexaol (12)

Off-white powder, 43%. ^1^H-NMR (500.20 MHz, DMSO, 25 °C): *δ* = 7.80 (s, 1H, H-9), 4.58 (t, *J*_OH15,15_ = 5.5 Hz, 1H, OH-15), 4.47 (t, *J*_10,11_ = 5.3 Hz, 2H, H-10), 4.41 (d, *J*_OH2,2_ = 5.5 Hz, 1H, OH-2), 4.34 (d, *J*_OH1,1_ = 5.8 Hz, 1H, OH-1), 4.26 (d, *J*_OH5,5_ = 7.8 Hz, 1H, OH-5), 4.21 (d, *J*_OH6,6_ = 7.5 Hz, 1H, OH-6), 4.11 (d, *J*_OH4,4_ = 7.2 Hz, 1H, OH-4), 4.04 (d, *J*_OH3,3_ = 7.7 Hz, 1H, OH-3), 3.98 (dddd, *J*_6,5_ = 1.4 Hz, *J*_6,7_ = 7.0 Hz, 1H, H-6), 3.80 (t, 2H, H-11), 3.72 (ddd, *J*_4,3_ < 1 Hz, *J*_4,5_ = 8.8 Hz, 1H, H-4), 3.61 (m, 1H, H-1a), 3.58 (ddd, *J*_3,2_ = 8.9 Hz, 1H, H-3), 3.52–3.49 (m, 4H, H-12, H-13), 3.47 (m, 2H, H-15), 3.46 (m, 1H, H-2), 3.39 (m, 2H, H-14), 3.38 (m, 1H, H-1b), 3.32 (ddd, under H_2_O signal, 1H, H-5), 2.79 (d, 2H, H-7) ppm. ^13^C-NMR (125.8 MHz, DMSO, 25 °C): *δ* = 145.0 (C-8), 123.1 (C-9), 72.3 (C-14), 71.6 (C-2), 70.9 (C-5), 69.9 (2 × C, C-12, C-13), 69.7 (C-3), 69.4 (C-6), 68.9 (C-11), 68.7 (C-4), 64.0 (C-1), 60.2 (C-15), 49.2 (C-10), 30.5 (C-7) ppm. HRMS calcd. for C_15_H_28_N_3_O_9_ [M − H]^−^: 394.1820, found: 394.1799.

#### (2*R*,3*R*,4*R*,5*R*,6*S*)-7-(1-(9*H*-Fluoren-9-yl)-1*H*-1,2,3-triazol-4-yl)heptane-1,2,3,4,5,6-hexaol (13)

Off-white powder, 49%. ^1^H-NMR (500.20 MHz, DMSO, 25 °C): *δ* = 7.97 (m, 2H, H-15, H-15′), 7.57 (s, 1H, H-9), 7.52 (m, 2H, H-14, H-14′), 7.40 (m, 2H, H-12, H-12′), 7.36 (m, 2H, H-13, H-13′), 6.93 (s, 1H, H-10), 4.38 (d, *J*_OH2,2_ = 5.6 Hz, 1H, OH-2), 4.32 (t, *J*_OH1,1_ = 5.7 Hz, 1H, OH-1) 4.25 (d, *J*_OH5,5_ = 7.7 Hz, 1H, OH-5), 4.17 (d, *J*_OH6,6_ = 7.7 Hz, 1H, OH-6), 4.08 (d, *J*_OH4,4_ = 7.3 Hz, 1H, OH-4), 4.00 (d, *J*_OH3,3_ = 7.8 Hz, 1H, OH-3), 3.93 (m, 1H, H-6), 3.69 (ddd, *J*_4,3_ = 1.4 Hz, *J*_4,5_ = 9.5 Hz, 1H, H-4), 3.61 (m, 1H, H-1a), 3.55 (ddd, *J*_3,2_ = 8.8 Hz, 1H, H-3), 3.46 (m, 1H, H-2), 3.37 (m, 1H, H-1b), 3.29 (ddd, *J*_5,6_ < 1 Hz, 1H, H-5), 2.77 (m, 2H, H-7) ppm. ^13^C-NMR (125.8 MHz, DMSO, 25 °C): *δ* = 145.4 (C-8), 141.5 (C-11), 140.0 (C-16), 129.4 (C-14), 128.0 (C-13), 125.0 (C-12), 121.2 (C-9), 120.6 (C-15), 71.4 (C-2), 70.9 (C-5), 69.6 (C-3), 69.1 (C-6), 68.6 (C-4), 63.8 (C-1), 63.4 (C-10), 30.4 (C-7) ppm. HRMS calcd. for C_22_H_25_N_3_O_6_Na [M − H]^−^: 450.1636, found: 450.1663.

### Single crystal X-ray analysis of compound 11

Single crystal analysis of crystals of compound 11 was carried out with a dual source (Cu/Mo) Agilent SuperNova diffractometer using monochromated (multilayer optics) Cu K_α_ radiation and Atlas CCD detector. The compound crystallizes as very small, plate-like crystal stacks aggregated in a fan-like formation. One tiny colorless crystal (30 × 50 × 100 μm) was mounted in a MiTeGen MicroMount™ and data collection was carried out at 120 K under N_2_ stream. The data was collected in *P*1 strategy but without separated Friedel pairs due the small crystal size that forced data collection to be 48 hours already with the mentioned strategy. Data collection, reduction and analytical numeric absorption corrections by multifaceted crystal models were all performed using CrysAlisPRO program.^11^ The crystal structure was solved with SHELXT^12^ and refined on *F*^2^ by full matrix least squares techniques with ShelXL^13^ program both implemented in Olex^2^ (v.1.2.10) program package.^14^ All non-hydrogen atoms were refined with anisotropic displacement parameters. Hydrogen atoms were treated as follows: C–H hydrogens were calculated into their ideal positions using isotropic displacement parameters 1.2 times of the host atom. All O–H atoms were first located on a difference Fourier map and at final stage refined as riding atoms. The lack of heavier scatterers and insufficient data quality prevented assignment of absolute configuration and the handedness was set based on the reference of the starting molecule. Atom occupancies on the disorder model of the phenyl ring were refined at first, but as the parameters settled very close to half they were fixed to 0.5 at the end of the refinement.

## Conclusions

To conclude, an improved method for preparation of propargylated d-mannose has been developed. This method provides access to larger quantities of the desired product, enabling more extensive utilization of this linear mannose-derived molecule in click reaction based applications. In addition, efficient synthesis protocols for mannose-derived sulfides and triazoles have been developed. Notably, based on ^1^H–^1^H NMR spectroscopic coupling constant analysis and single crystal X-ray diffraction, the linear conformation of the polyol backbone remains unaffected by the chain-elongation, indicating that these mannose derived polyols could be used as stiff, hydrogen bond-forming building blocks in polymer and other material science applications.

Future studies will focus on further application of the developed protocols by covalently linking the mannose-derivatives to larger molecular architectures, such as polymers, dendrimers, silica and other surfaces.

## Conflicts of interest

There are no conflicts to declare.

## Supplementary Material

RA-010-C9RA10378C-s001

RA-010-C9RA10378C-s002
