# Supplementary material for: Improved synthesis and application of conjugation-amenable polyols from d-mannose
Source: RSC Adv. 2020 Jan 23;10(7):3960–6. doi: 10.1039/c9ra10378c (PMC9048844; doi:10.1039/c9ra10378c)
Supplement: RA-010-C9RA10378C-s001 [file RA-010-C9RA10378C-s001.pdf]

Electronic Supplementary Material (ESI) for RSC Advances.
This journal is © The Royal Society of Chemistry 2020

Supporting information for

Improved synthesis and application of conjugation-amenable polyols from D-mannose

Ida Mattsson, Ruzal Sitdikov, Andreas C. M. Gunell, Tiina Saloranta-Simell, Manu Lahtinen and Reko Leino*

Contents

Synthesis and NMR data of (2*R*,3*R*,4*R*,5*R*,6*S*)-non-8-ene-1,2,3,4,5,6-hexaol (**1**) 2

Optimization of synthesis of (2*R*,3*R*,4*R*,5*R*,6*S*)-non-8-yne-1,2,3,4,5,6-hexaol (**2**) 3

NMR spectra of (2*R*,3*R*,4*R*,5*R*,6*S*)-non-8-yne-1,2,3,4,5,6-hexaol (**2**) 4

Synthesis and NMR spectra of (2*R*,3*R*,4*R*,5*R*,6*S*)-9-(propylthio)nonane-1,2,3,4,5,6-hexaol (**3**) 5

Synthesis and NMR spectra of (2*R*,2'*R*,3*R*,3'*R*,4*R*,4'*R*,5*R*,5'*R*,6*S*,6'*S*)-9,9'-(propane-1,3-diylbis(sulfanediyl))bis(nonane-1,2,3,4,5,6-hexaol) (**4**) 7

Synthesis and NMR spectra of (2*R*,3*R*,4*R*,5*R*,6*S*)-9-(benzylthio)nonane-1,2,3,4,5,6-hexaol (**5**) 9

Synthesis and NMR spectra of *S*-((4*S*,5*R*,6*R*,7*R*,8*R*)-4,5,6,7,8,9-hexahydroxynonyl)-L-cysteine (**6**) . 11

Synthesis and NMR spectra of (2*R*,3*R*,4*R*,5*R*,6*S*)-9-(cyclohexylthio)nonane-1,2,3,4,5,6-hexaol (**7**). 13

Synthesis and NMR spectra of (2*R*,3*R*,4*R*,5*R*,6*S*)-9-((2-(2-(2-hydroxyethoxy)ethoxy)ethyl)thio)nonane-1,2,3,4,5,6-hexaol (**8**) 15

Synthesis and NMR spectra of 2,2-bis(((3-(((4*S*,5*R*,6*R*,7*R*,8*R*)-4,5,6,7,8,9 hexahydroxynonyl)thio)propanoyl)oxy)methyl)propane-1,3-diylbis(3-(((4*S*,5*R*,6*R*,7*R*,8*R*)-4,5,6,7,8,9-hexahydroxynonyl)thio)propanoate) (**9**) 17

UV-Reactor setup 19

Synthesis and NMR spectra of (2*R*,3*R*,4*R*,5*R*,6*S*)-7-(1-(2-hydroxyethyl)-1*H*-1,2,3-triazol-4-yl)heptane-1,2,3,4,5,6-hexaol (**10**) 20

Synthesis and NMR spectra of (2*R*,3*R*,4*R*,5*R*,6*S*)-7-(1-benzyl-1*H*-1,2,3-triazol-4-yl)- heptane-1,2,3,4,5,6-hexaol (**11**) 22

Synthesis and NMR spectra of (2*R*,3*R*,4*R*,5*R*,6*S*)-7-(1-(2-(2-(2-hydroxyethoxy)ethoxy)ethyl)-1*H*-1,2,3-triazol-4-yl)heptane-1,2,3,4,5,6-hexaol (**12)** 24

Synthesis and NMR spectra of (2*R*,3*R*,4*R*,5*R*,6*S*)-7-(1-(9*H*-fluoren-9-yl)-1H-1,2,3-triazol-4-yl)heptane-1,2,3,4,5,6-hexaol (**13**) 26

Crystal data of (2*R*,3*R*,4*R*,5*R*,6*S*)-7-(1-benzyl-1*H*-1,2,3-triazol-4-yl)- heptane-1,2,3,4,5,6-hexaol (**11**) 28

Synthesis and NMR data of (2*R*,3*R*,4*R*,5*R*,6*S*)-non-8-ene-1,2,3,4,5,6-hexaol (**1**)

D-Mannose (5 g, 27.8 mmol, 1 eq), tin powder (6.7 g, 56.4 mmol, 2 eq) and allyl bromide (10 g, 83.2 mmol, 3 eq) were dissolved in 550 ml EtOH and 50 ml distilled H_2O. The reaction mixture was heated to 60 °C and was stirred overnight. The mixture was allowed to cool to room temperature and was neutralized with 5 M NaOH (aq). The mixture was filtered through Celite. The filtrate was evaporated until approximately 80 ml solution remained. The solution was left in frigde overnight to yield 2.96 g white crystals (48 % yield).

^{1}H-NMR (500.20 MHz, DMSO, 25 °C): δ = 5.84 (dddd, $J_{8,7a}$ = 6.6 Hz, $J_{8,7b}$ = 7.5 Hz, $J_{8,9trans}$ = 17.2 Hz, $J_{8,9cis}$ = 10.2 Hz, 1 H, H-8), 5.04 (dddd, $J_{9trans,7a}$ = -1.5 Hz, $J_{9trans,7b}$ = -1.4 Hz, $J_{9trans,9cis}$ = -2.4 Hz, 1 H, H-9trans), 4.98 (dddd, $J_{9cis,7a}$ = 1.1 Hz, $J_{9cis,7b}$ = 1.2 Hz, 1 H, H-9cis), 4.38 (d, $J_{OH2,2}$ = 5.6 Hz, 1 H, OH-2), 4.32 (t, $J_{OH1,1a}$ = $J_{OH2,2}$ = 5.6 Hz, 1 H, OH-1), 4.09 (d, $J_{OH4,4}$ = 7.2 Hz, 1 H, OH-4), 4.08 (d, $J_{OH5,5}$ = 7.2 Hz, 1 H, OH-5), 4.08 (d, $J_{OH6,6}$ = 7.8 Hz, 1 H, OH-6), 4.03 (d, $J_{OH3,3}$ = 7.7 Hz, 1 H, OH-3), 3.74 (dddd, $J_{6,5}$ = 1.6 Hz, $J_{6,7a}$ = 7.7 Hz, $J_{6,7b}$ = 5.9 Hz, 1 H, H-6), 3.96 (ddd, $J_{4,3}$ < 0.5 Hz, $J_{4,5}$ = 9.3 Hz, 1 H, H-4), 3.61 (ddd, $J_{1a,1b}$ = -11.0 Hz, $J_{1a,2}$ = 3.6 Hz, 1 H, H-1a), 3.56 (ddd, $J_{3,2}$ = 8.3 Hz, 1 H, H-3), 3.47 (dddd, $J_{2,1b}$ = 6.1 Hz, 1 H, H-2), 3.38 (ddd, 1 H, H-1b), 3.29 (ddd, 1 H, H-5), 2.23 (ddddd, $J_{7a,7b}$ = -14.0 Hz, 1 H, H-7a), 2.20 (ddddd, 1 H, H-7b) ppm.

^{13}C-NMR (125.8 MHz, DMSO, 25 °C): δ = 137.2 (C-8), 116.4 (C-9), 72.0 (C-2), 71.4 (C-5), 70.2 (C-3), 69.8 (C-6), 69.1 (C-4), 64.4 (C-1), 39.0 (C-7).

Optimization of synthesis of (2*R*,3*R*,4*R*,5*R*,6*S*)-non-8-yne-1,2,3,4,5,6-hexaol (2)

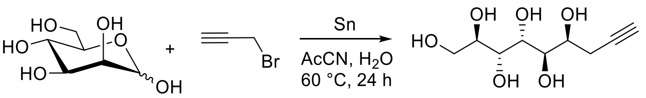

Table S1. Investigated optimization parameters.

Entry	Deviation from standard conditions[a]	Conversion of mannose[b] (%)
1	None	69
2	Reaction temperature 80 °C	13
3	Reaction temperature 65 °C	24
4	Indium powder instead of Sn, reaction temperature 80 °C	77 [c]
5	4 days reaction time at room temperature	57
6	K_2CO_3 and $SnBu_2(O)$ instead of Sn powder	No reaction
7	1 eq. of PrBr, then 1 eq. added after 12 h.	39
8	Solvent EtOH/H_2O (9/1)	No reaction
9	Reaction conducted at room temperature	10
10	Solvent DMF	24
11	Solvent THF/H_2O (9/1)	No reaction
12	Solvent EtOH/H_2O (9.5/0.5), reaction temperature 40 °C	1
13	Solvent EtOH/H_2O (9.5/0.5), reaction temperature 50 °C	9

[a] Standard conditions: Sn powder, AcCN/H_2O 9/1, 60 °C, 24 h; [b] Determined by ^{1}H NMR spectroscopy; [c] Increased side product formation, separation difficulties.

NMR spectra of (2*R*,3*R*,4*R*,5*R*,6*S*)-non-8-yne-1,2,3,4,5,6-hexaol (**2**)

^{1}H-NMR spectrum of (2*R*,3*R*,4*R*,5*R*,6*S*)-non-8-yne-1,2,3,4,5,6-hexaol (**2**)

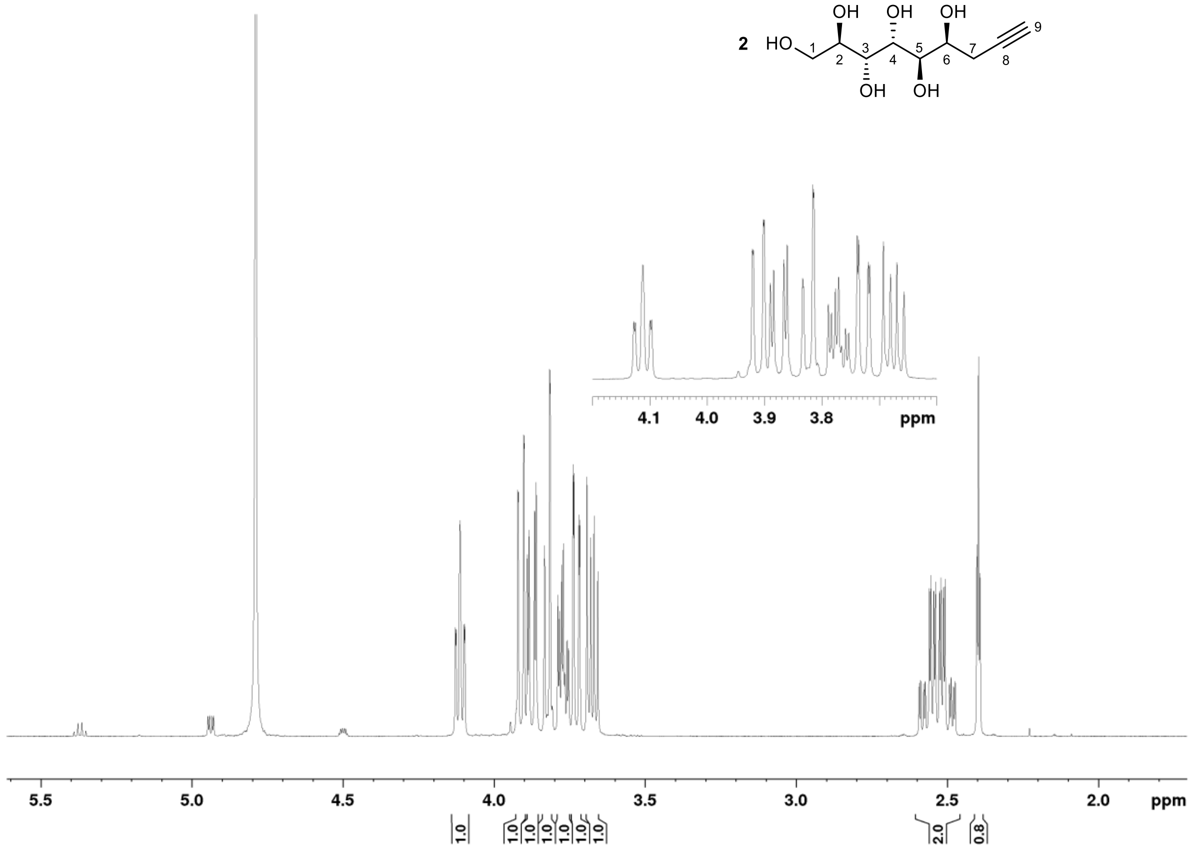

^{13}C-NMR spectrum of (2*R*,3*R*,4*R*,5*R*,6*S*)-non-8-yne-1,2,3,4,5,6-hexaol (**2**)

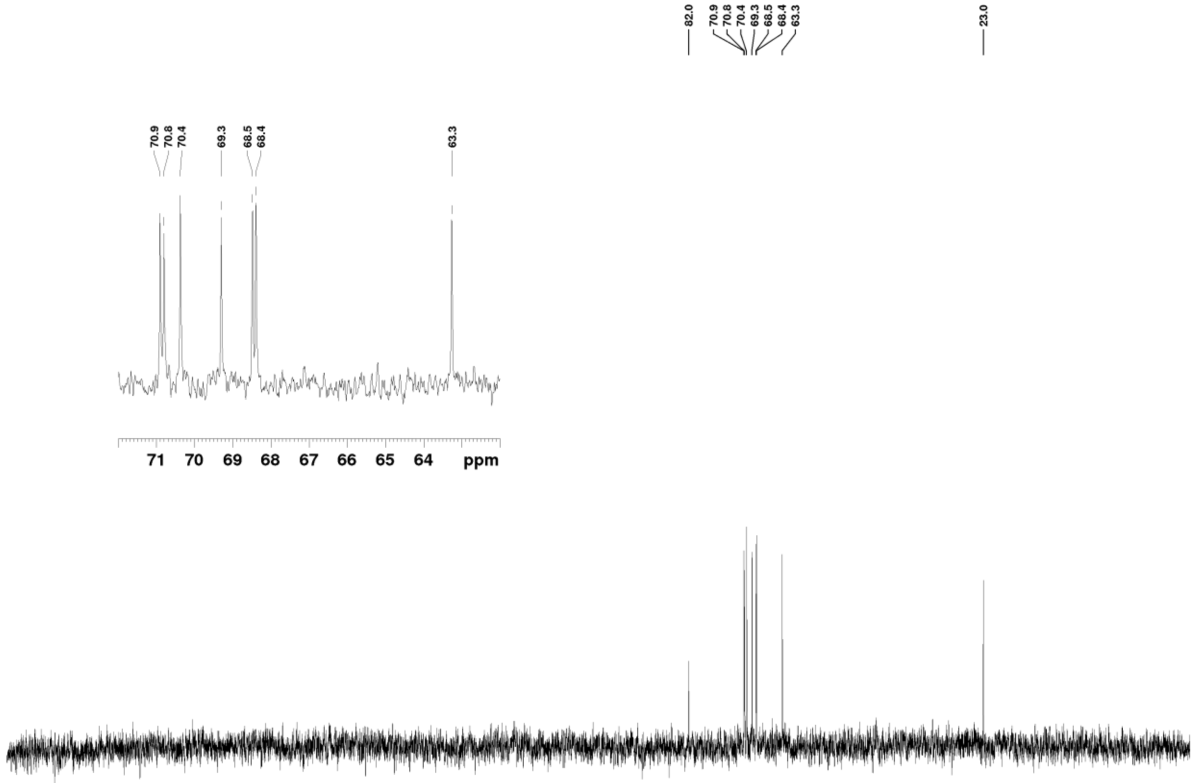

Synthesis and NMR spectra of (2*R*,3*R*,4*R*,5*R*,6*S*)-9-(propylthio)nonane-1,2,3,4,5,6-hexaol (**3**)

3

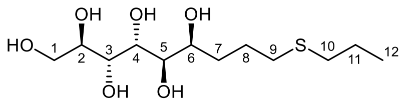

Allylated D-mannose (100 mg, 0.45 mmol, 1 eq.), propanethiol (84 µl, 0.9 mmol, 2 eq.) and 2,2-dimethoxy-2-phenylacetophenone (5.8 mg, 0.023 mmol, 0.05 eq.) were dissolved in 10 ml MeOH:H_2O 1:1. The reaction mixture was irradiated with 365 nm UV-light for 1 h. The mixture was subsequently evaporated to dryness and the solids were stirred in 3 ml hexane for 1 h before centrifugation, decantation and drying *in vacuo*. The reaction yielded 125 mg of off-white powder (93 %).

^{1}H-NMR spectrum of (2*R*,3*R*,4*R*,5*R*,6*S*)-9-(propylthio)nonane-1,2,3,4,5,6-hexaol (**3**)

3

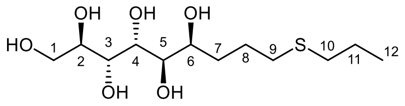

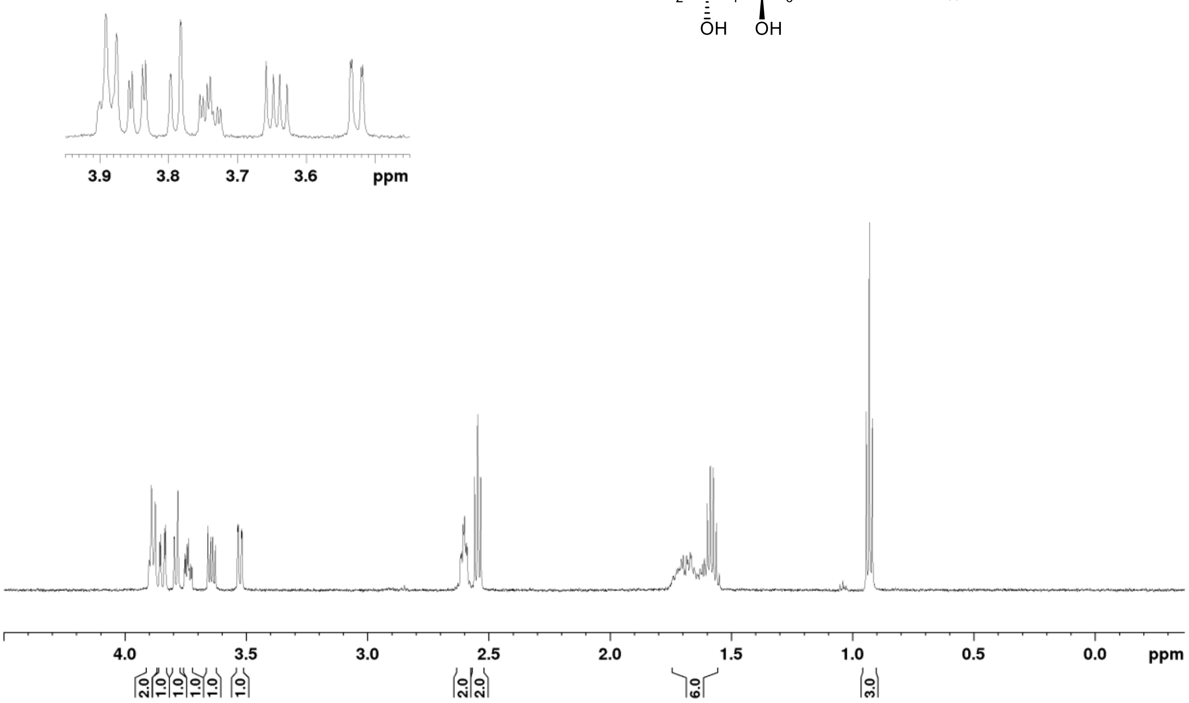

^{13}C-NMR spectrum of compound (2*R*,3*R*,4*R*,5*R*,6*S*)-9-(propylthio)nonane-1,2,3,4,5,6-hexaol (**3**)

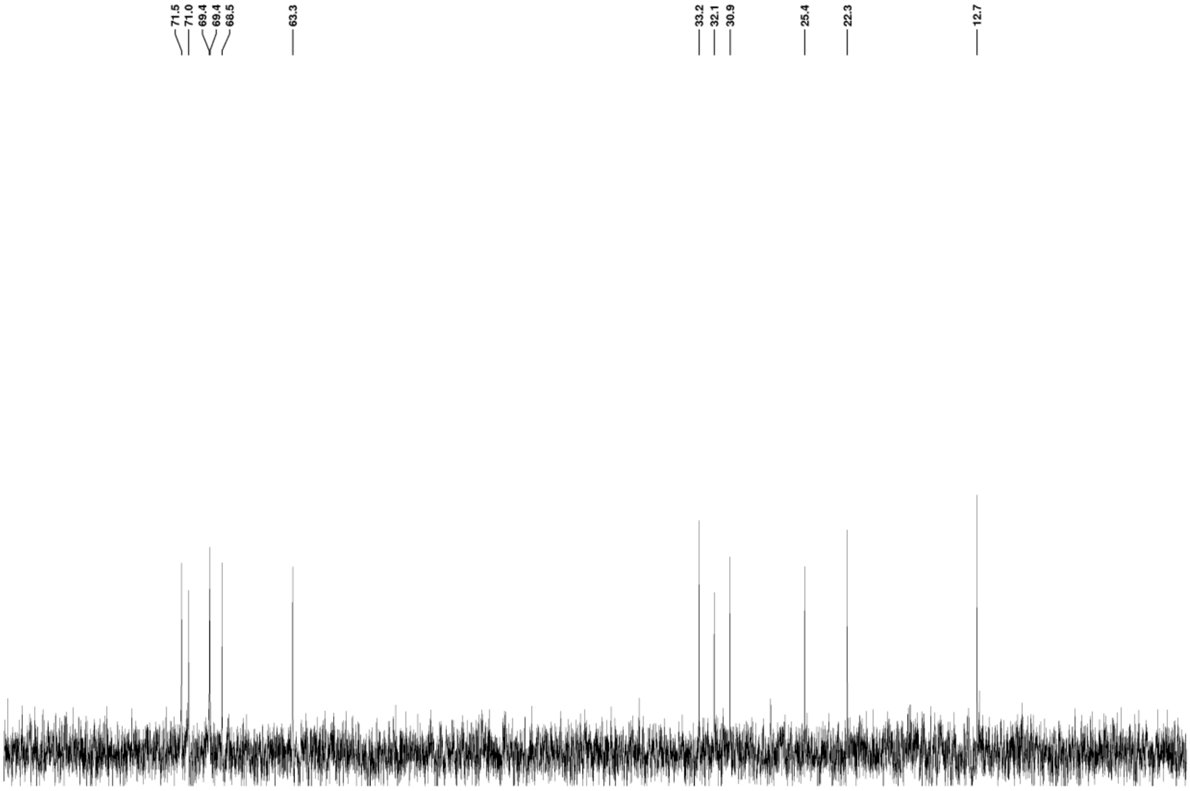

Synthesis and NMR spectra of (2*R*,2'*R*,3*R*,3'*R*,4*R*,4'*R*,5*R*,5'*R*,6*S*,6'*S*)-9,9'-(propane-1,3-diylbis(sulfanediyl))bis(nonane-1,2,3,4,5,6-hexaol) (**4**)

4 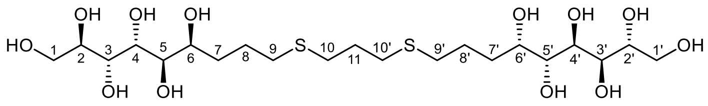

Allylated D-mannose (100 mg, 0.45 mmol, 2.1 eq.), propanedithiol (22 µl, 0.215 mmol, 1 eq.) and 2,2-dimethoxy-2-phenylacetophenone (5.5 mg, 0.0215 mmol, 0.1 eq.) were dissolved in 10 ml MeOH:H_2O 1:1. The reaction mixture was irradiated with 365 nm UV-light for 1 h. The mixture was subsequently evaporated to dryness and the solids were stirred in 2 ml water to remove excess allylated D-mannose for 30 min before centrifugation, decantation and drying *in vacuo*. The reaction yielded 101 mg of off-white powder (85 %).

^{1}H-NMR spectrum of (2*R*,2'*R*,3*R*,3'*R*,4*R*,4'*R*,5*R*,5'*R*,6*S*,6'*S*)-9,9'-(propane-1,3-diylbis(sulfanediyl)) bis(nonane-1,2,3,4,5,6-hexaol) (**4**)

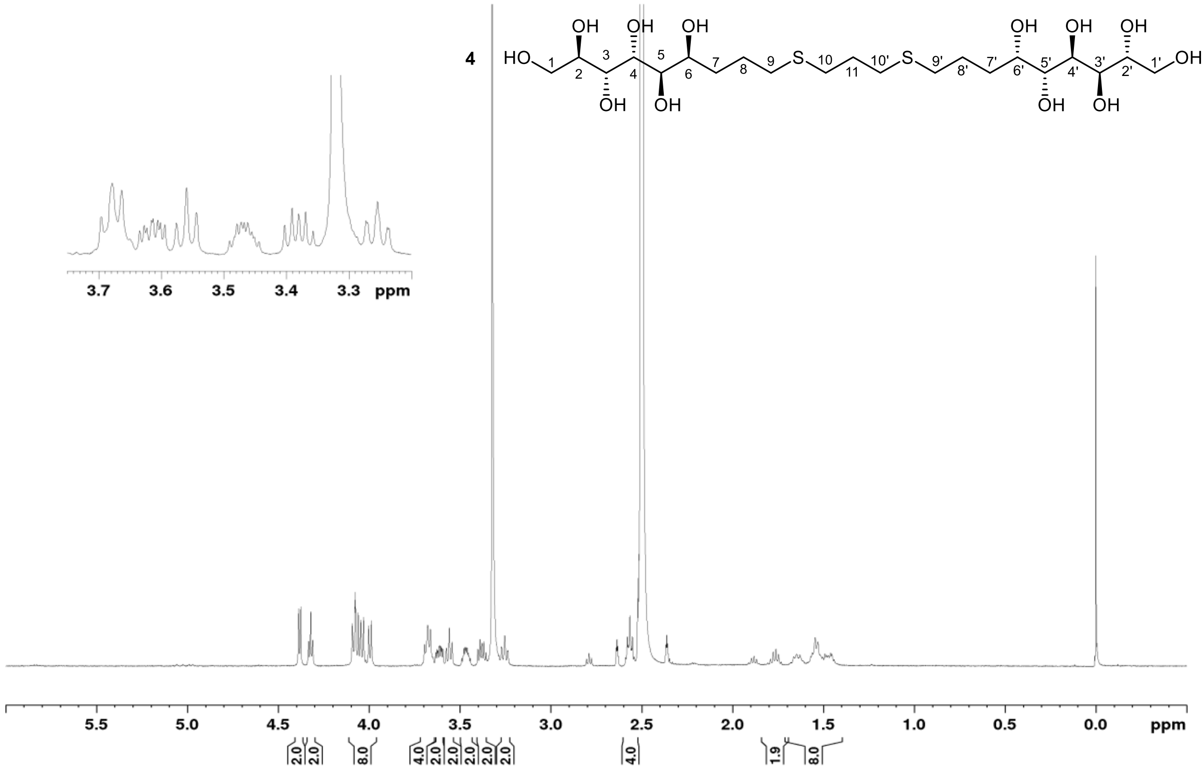

^{13}C-NMR spectrum of (2*R*,2'*R*,3*R*,3'*R*,4*R*,4'*R*,5*R*,5'*R*,6*S*,6'*S*)-9,9'-(propane-1,3-diylbis(sulfanediyl)) bis(nonane-1,2,3,4,5,6-hexaol) (**4**)

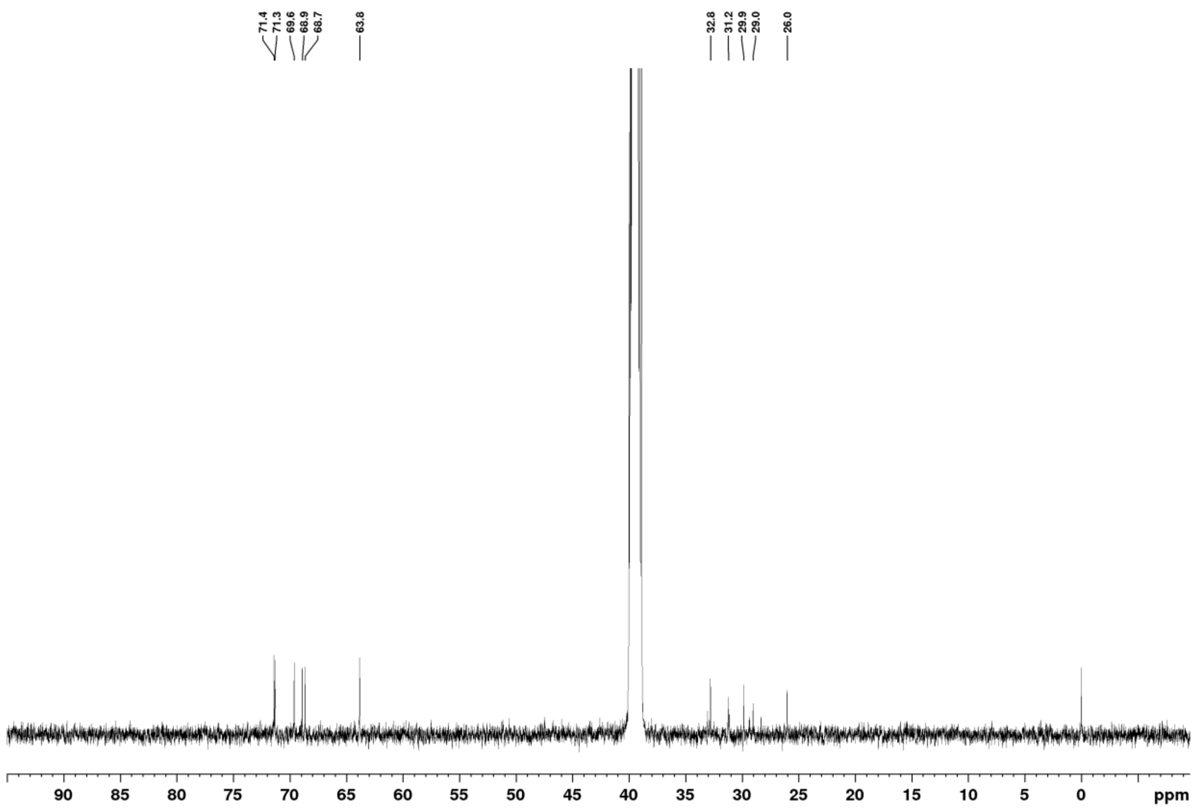

Synthesis and NMR spectra of (2*R*,3*R*,4*R*,5*R*,6*S*)-9-(benzylthio)nonane-1,2,3,4,5,6-hexaol (**5**)

5

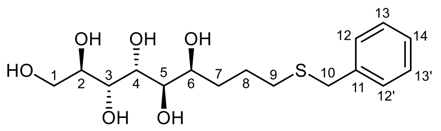

Allylated D-mannose (100 mg, 0.45 mmol, 1 eq.), phenylmethanethiol (105 µl, 0.9 mmol, 2 eq.) and 2,2-dimethoxy-2-phenylacetophenone (5.8 mg, 0.023 mmol, 0.05 eq.) were dissolved in 10 ml MeOH:H_2O 1:1 and 4 ml DMF in order to improve solubility of the thiol. The reaction mixture was irradiated with 365 nm UV-light for 1 h. The mixture was subsequently evaporated to dryness and the solids were stirred in 3 ml toluene for 1 h before centrifugation, decantation and drying *in vacuo*. The reaction yielded 126 mg of off-white powder (81 %).

^{1}H-NMR spectrum of (2*R*,3*R*,4*R*,5*R*,6*S*)-9-(benzylthio)nonane-1,2,3,4,5,6-hexaol (**5**)

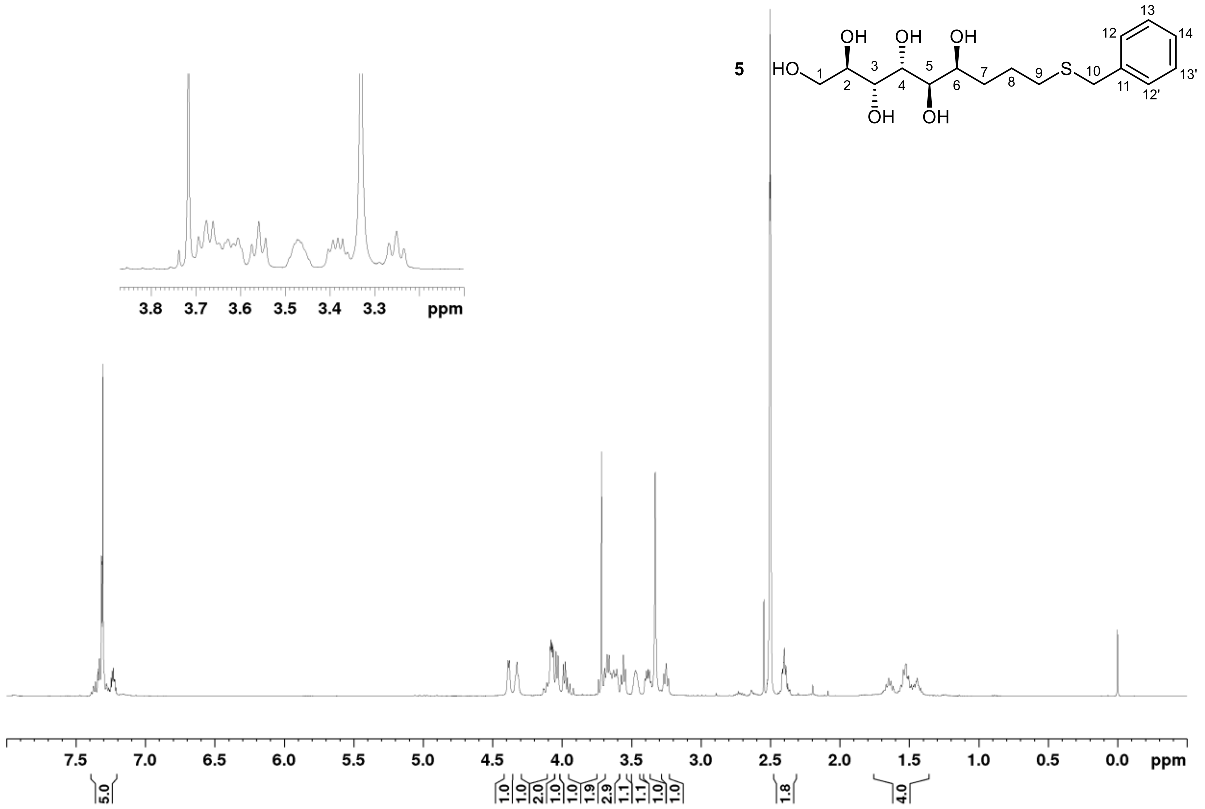

^{13}C-NMR spectrum of (2*R*,3*R*,4*R*,5*R*,6*S*)-9-(benzylthio)nonane-1,2,3,4,5,6-hexaol (**5**)

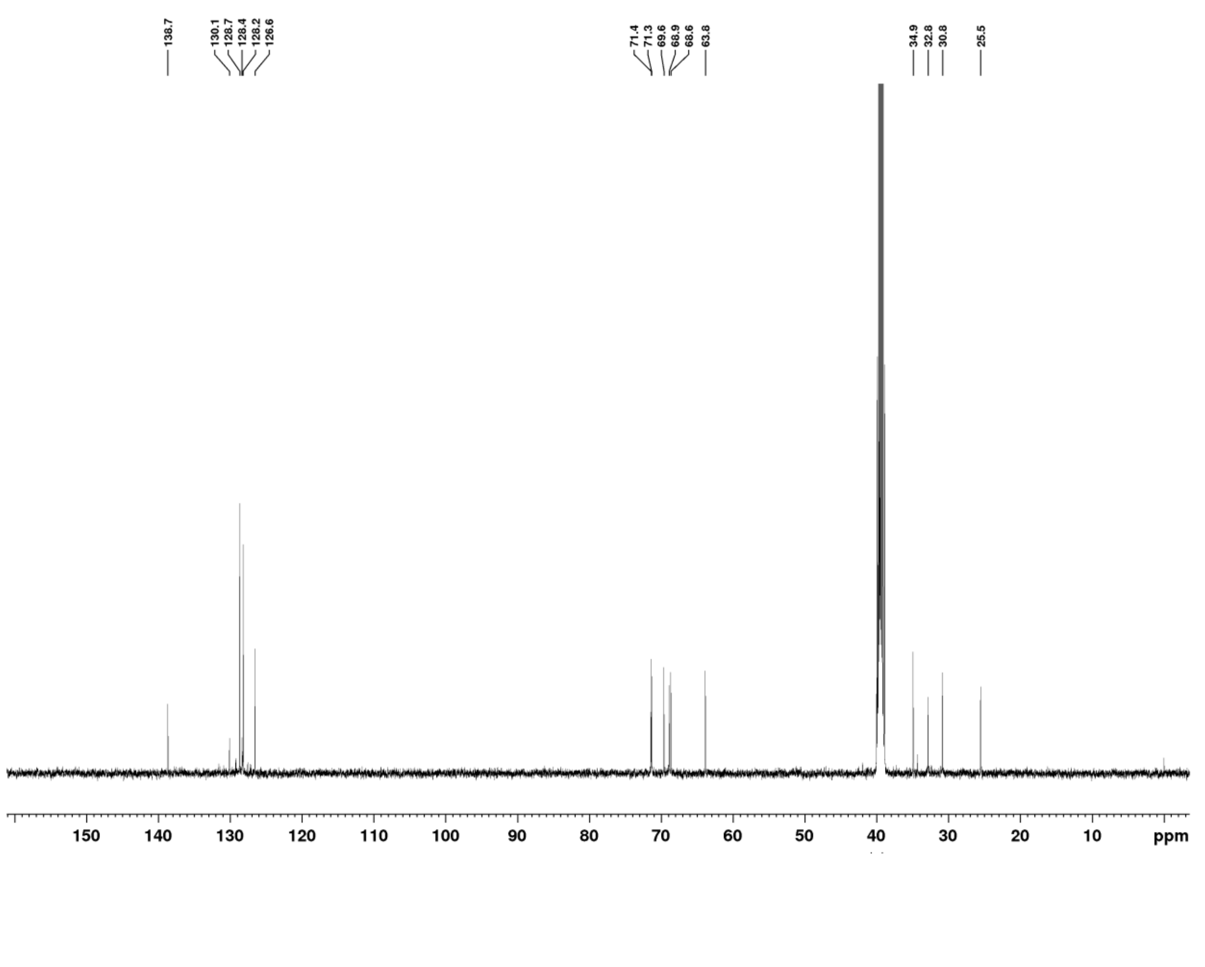

Synthesis and NMR spectra of *S*-((4*S*,5*R*,6*R*,7*R*,8*R*)-4,5,6,7,8,9-hexahydroxynonyl)-L-cysteine (**6**)

6 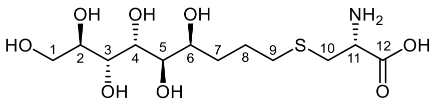

Allylated D-mannose (100 mg, 0.45 mmol, 1 eq.), L-cysteine (55 mg, 0.45 mmol, 1 eq.) and 2,2-dimethoxy-2-phenylacetophenone (5.8 mg, 0.023 mmol, 0.05 eq.) were dissolved in 10 ml MeOH:H_2O 1:1. The reaction mixture was irradiated with 365 nm UV-light for 1 h. The mixture was subsequently evaporated to dryness and the solids were stirred in 3 ml acetone before centrifugation and decantation. The washing procedure was repeated with 0.5 ml distilled water, followed by centrifugation, decantation and drying *in vacuo*. The reaction yielded 119 mg of white powder (77 %).

^{1}H-NMR spectrum of *S*-((4*S*,5*R*,6*R*,7*R*,8*R*)-4,5,6,7,8,9-hexahydroxynonyl)-L-cysteine (**6**)

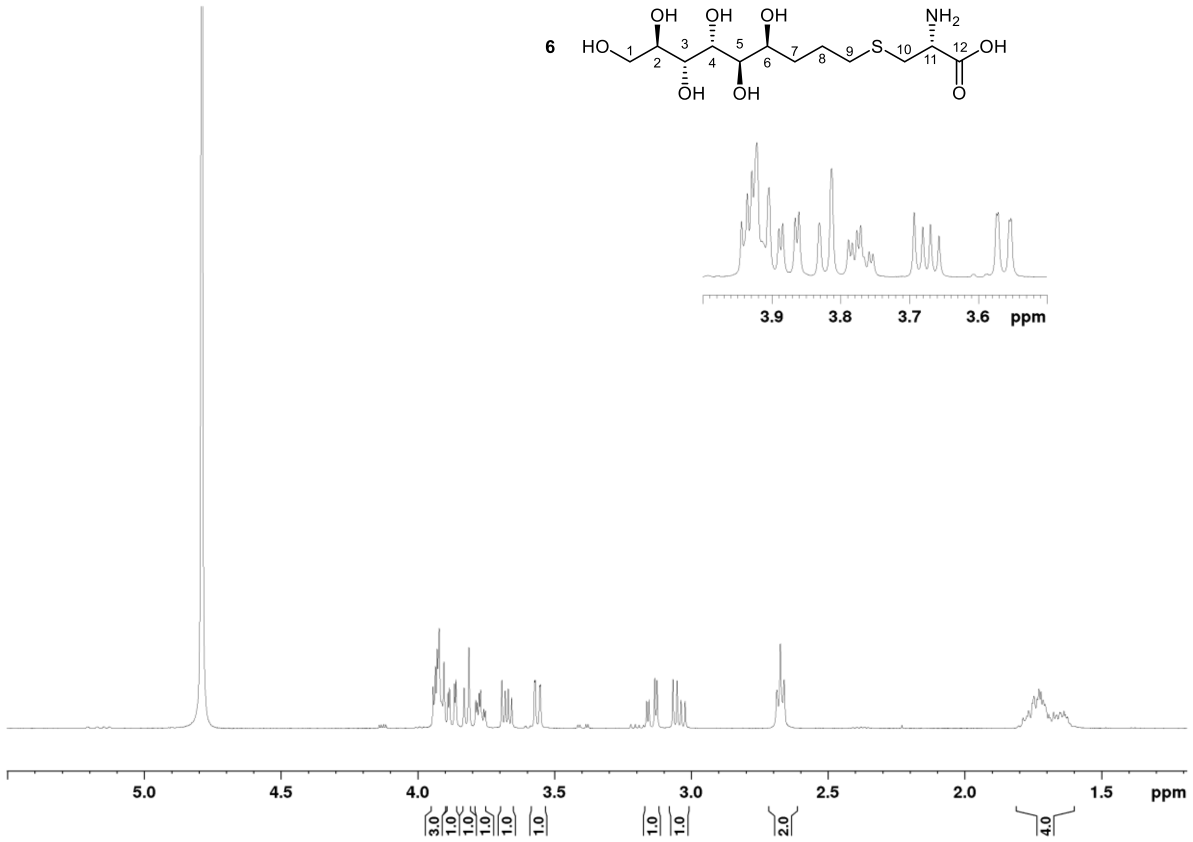

^{13}C-NMR spectrum of *S*-((4*S*,5*R*,6*R*,7*R*,8*R*)-4,5,6,7,8,9-hexahydroxynonyl)-L-cysteine (**6**)

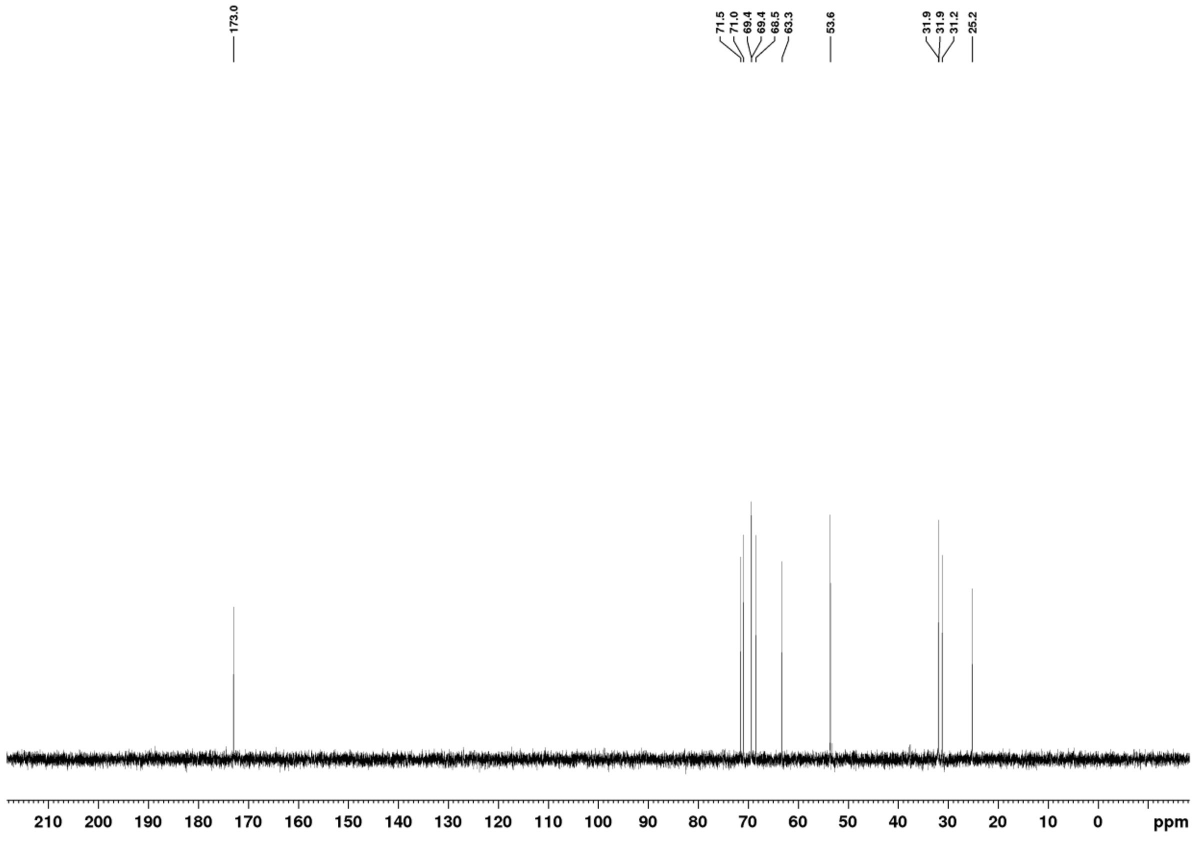

Synthesis and NMR spectra of (2*R*,3*R*,4*R*,5*R*,6*S*)-9-(cyclohexylthio)nonane-1,2,3,4,5,6-hexaol (7)

7

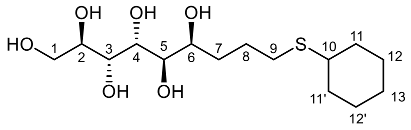

Allylated D-mannose (100 mg, 0.45 mmol, 1 eq.), cyclohexanethiol (110 µl, 0.9 mmol, 2 eq.) and 2,2-dimethoxy-2-phenylacetophenone (5.8 mg, 0.023 mmol, 0.05 eq.) were dissolved in 10 ml MeOH:H_2O 1:1 and 4 ml DMF in order to improve solubility of the thiol. The reaction mixture was irradiated with 365 nm UV-light for 1 h. The mixture was subsequently evaporated to dryness and the solids were stirred in 3 ml hexane for 1 h before centrifugation, decantation and drying *in vacuo*. The reaction yielded 138 mg of off-white powder (91 %).

^{1}H-NMR spectrum of (2*R*,3*R*,4*R*,5*R*,6*S*)-9-(cyclohexylthio)nonane-1,2,3,4,5,6-hexaol (**7**)

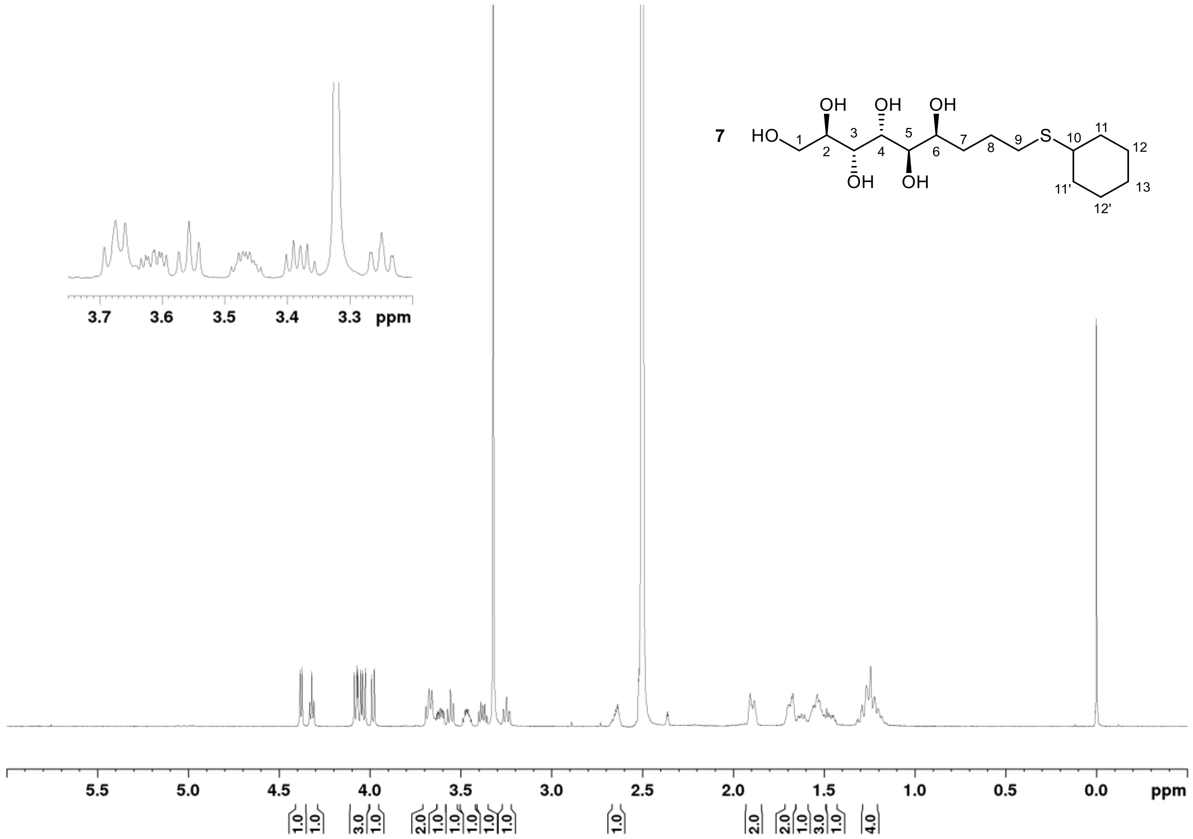

^{13}C-NMR spectrum of (2*R*,3*R*,4*R*,5*R*,6*S*)-9-(cyclohexylthio)nonane-1,2,3,4,5,6-hexaol (**7**)

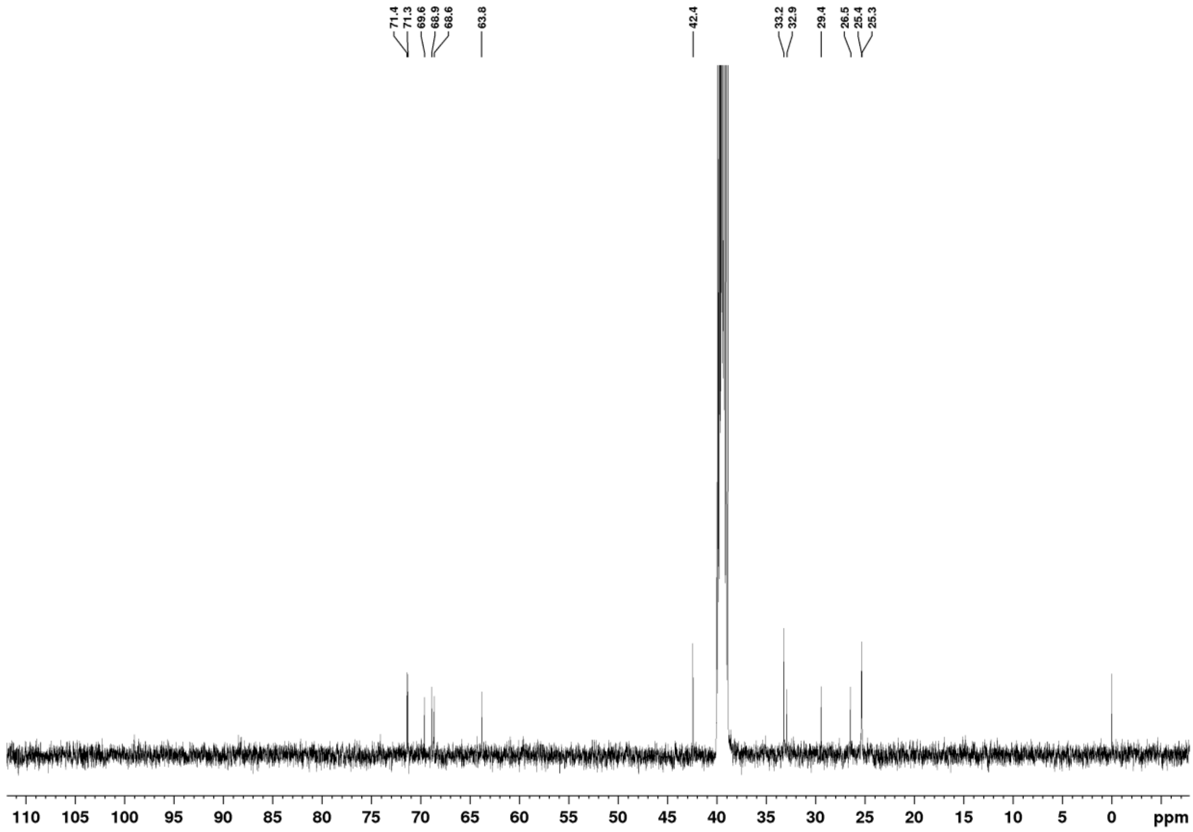

Synthesis and NMR spectra of (2*R*,3*R*,4*R*,5*R*,6*S*)-9-((2-(2-(2-hydroxyethoxy)ethoxy)ethyl)thio)nonane-1,2,3,4,5,6-hexaol (**8**)

8

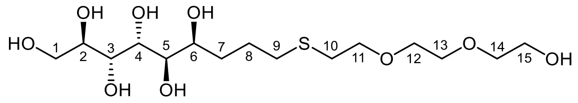

Allylated D-mannose (100 mg, 0.45 mmol, 1 eq.), 2-[2-(2-mercaptoethoxy)ethoxy]ethanol (150 mg, 0.9 mmol, 2 eq.) and 2,2-dimethoxy-2-phenylacetophenone (5.8 mg, 0.023 mmol, 0.05 eq.) were dissolved in 10 ml MeOH:H_2O 1:1. The reaction mixture was irradiated with 365 nm UV-light for 1 h. The mixture was subsequently evaporated to dryness and the solids were stirred in 3 ml ethyl acetate for 1 h before centrifugation, decantation and drying *in vacuo*. The reaction yielded 198 mg of off-white powder (65 %).

^{1}H-NMR spectrum of (2*R*,3*R*,4*R*,5*R*,6*S*)-9-((2-(2-(2-hydroxyethoxy)ethoxy)ethyl)thio)nonane-1,2,3,4,5,6-hexaol (**8**)

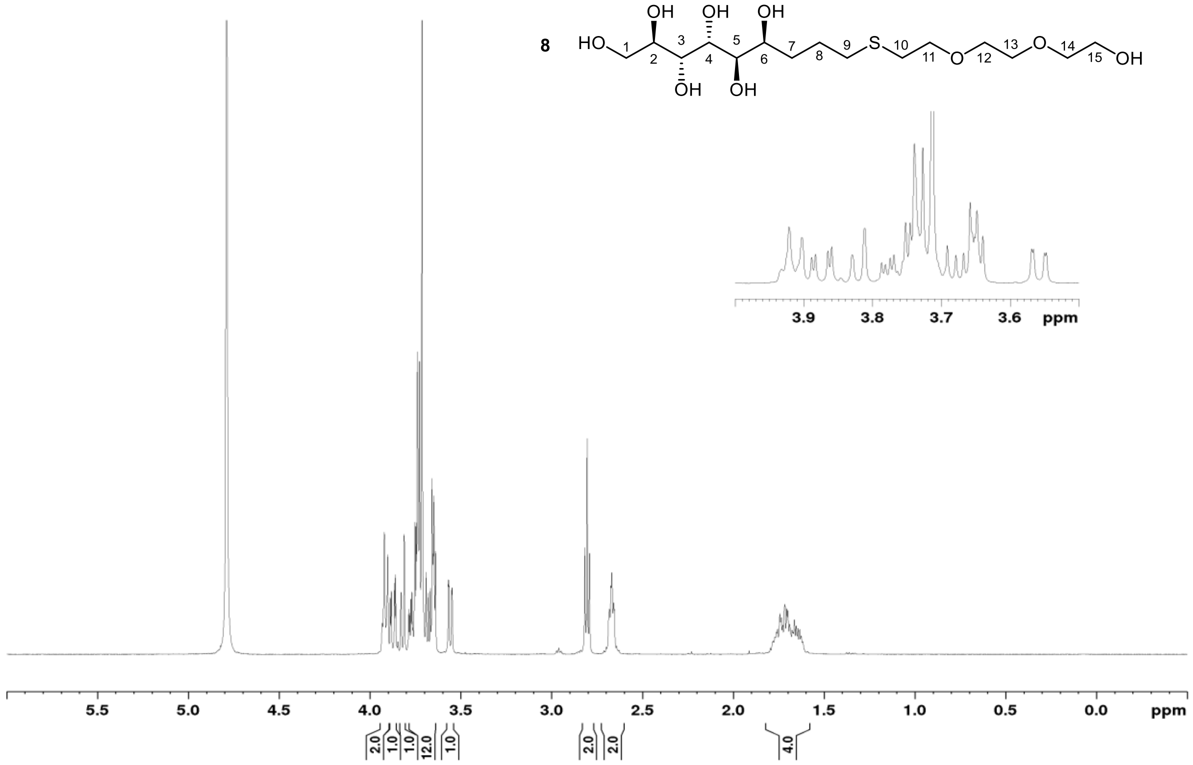

^{13}C-NMR spectrum of (2*R*,3*R*,4*R*,5*R*,6*S*)-9-((2-(2-(2-hydroxyethoxy)ethoxy)ethyl)thio)nonane-1,2,3,4,5,6-hexaol (**8**)

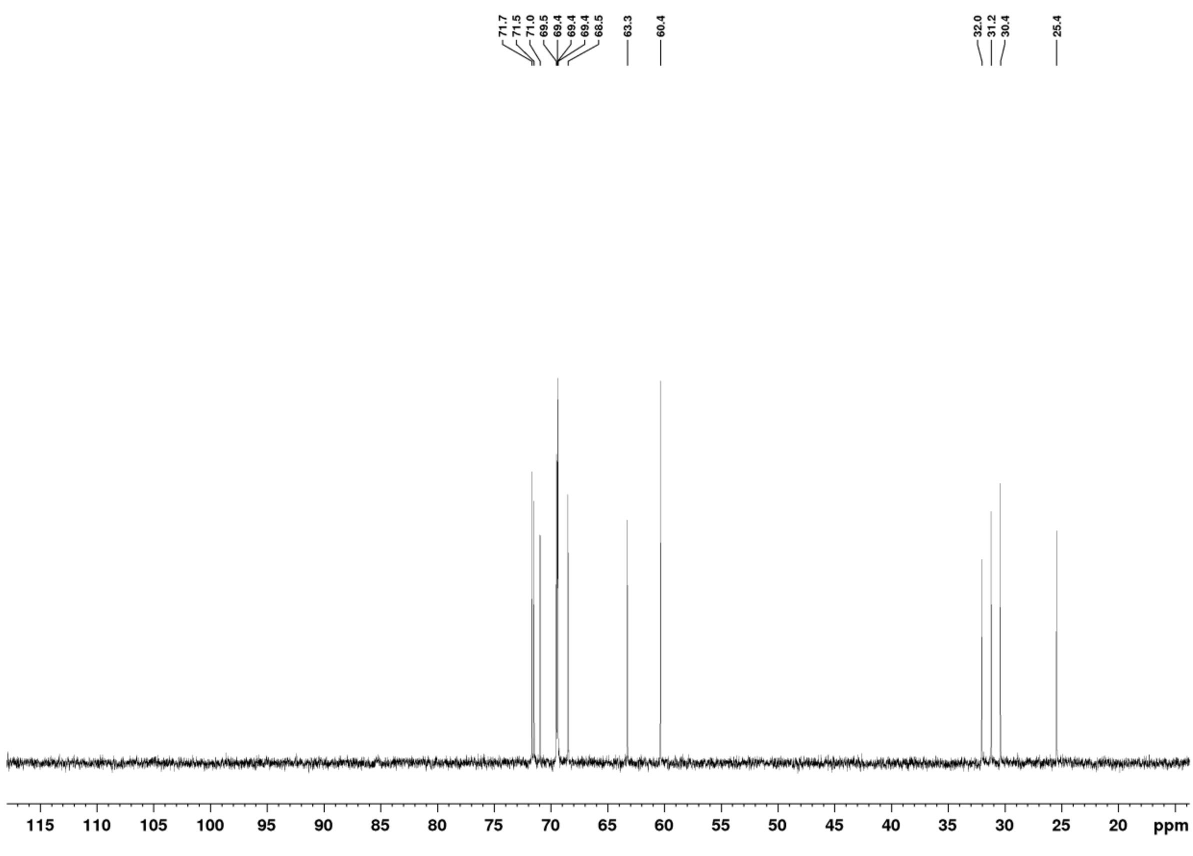

Synthesis and NMR spectra of 2,2-bis(((3-(((4*S*,5*R*,6*R*,7*R*,8*R*)-4,5,6,7,8,9 hexahydroxynonyl)thio)propanoyl)oxy)methyl)propane-1,3-diylbis(3-(((4*S*,5*R*,6*R*,7*R*,8*R*)-4,5,6,7,8,9-hexahydroxynonyl)thio)propanoate) (**9**)

9

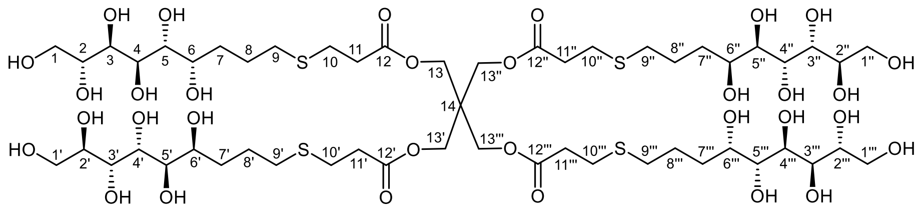

Allylated D-mannose (100 mg, 0.45 mmol, 5 eq.), pentaerythritol tetrakis(3-mercaptopropionate) (44 mg, 0.09 mmol, 1 eq.) and 2,2-dimethoxy-2-phenylacetophenone (5.8 mg, 0.023 mmol, 0.25 eq.) were dissolved in 10 ml MeOH:H_2O 1:1. The reaction mixture was irradiated with 365 nm UV-light for 1 h. The mixture was subsequently evaporated to dryness and the solids were stirred in 3 ml ethyl acetate and 1 ml distilled water for 1 h before centrifugation and decantation and drying *in vacuo*. The reaction yielded 90 mg of off-white powder (73 %).

^{1}H-NMR spectrum of 2,2-bis(((3-(((4*S*,5*R*,6*R*,7*R*,8*R*)-4,5,6,7,8,9 hexahydroxynonyl)thio)propanoyl)oxy)methyl)propane-1,3-diylbis(3-(((4*S*,5*R*,6*R*,7*R*,8*R*)-4,5,6,7,8,9-hexahydroxynonyl)thio)propanoate) (**9**)

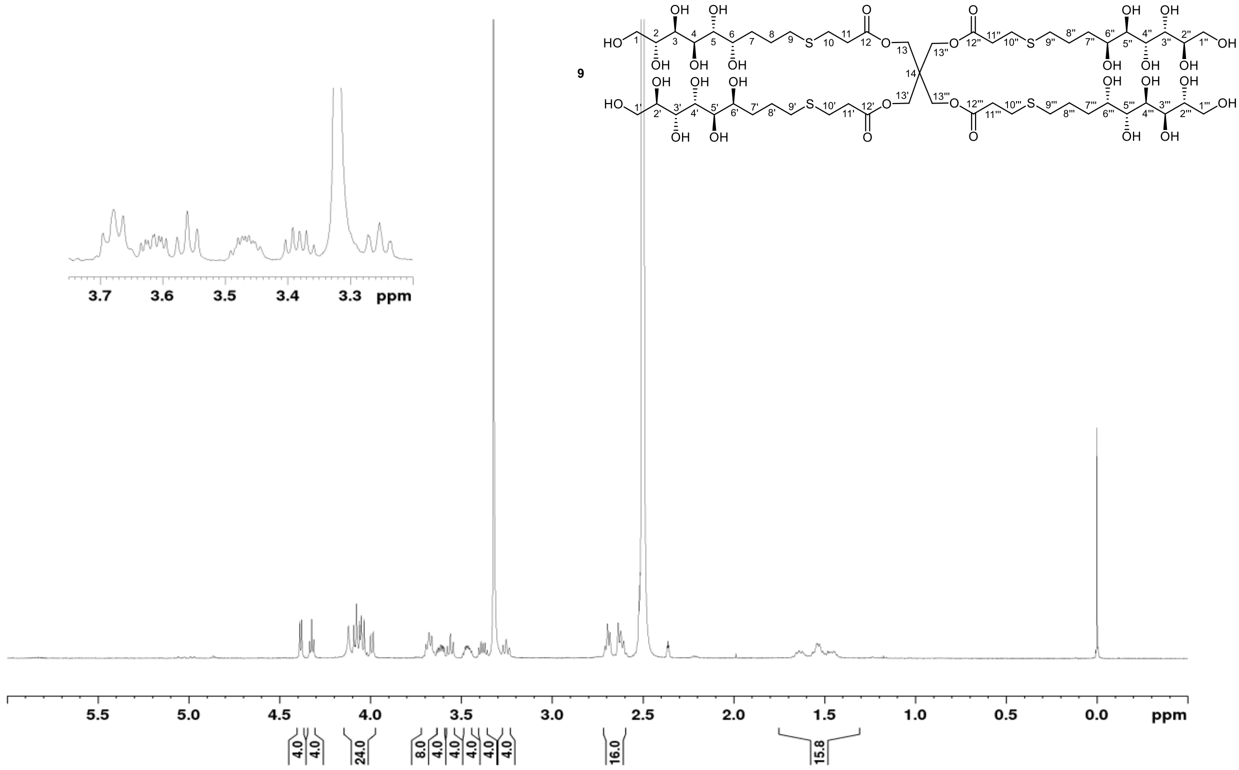

^{13}C-NMR spectrum of 2,2-bis(((3-(((4*S*,5*R*,6*R*,7*R*,8*R*)-4,5,6,7,8,9 hexahydroxynonyl)thio)propanoyl)oxy)methyl)propane-1,3-diylbis(3-(((4*S*,5*R*,6*R*,7*R*,8*R*)-4,5,6,7,8,9-hexahydroxynonyl)thio)propanoate) (**9**)

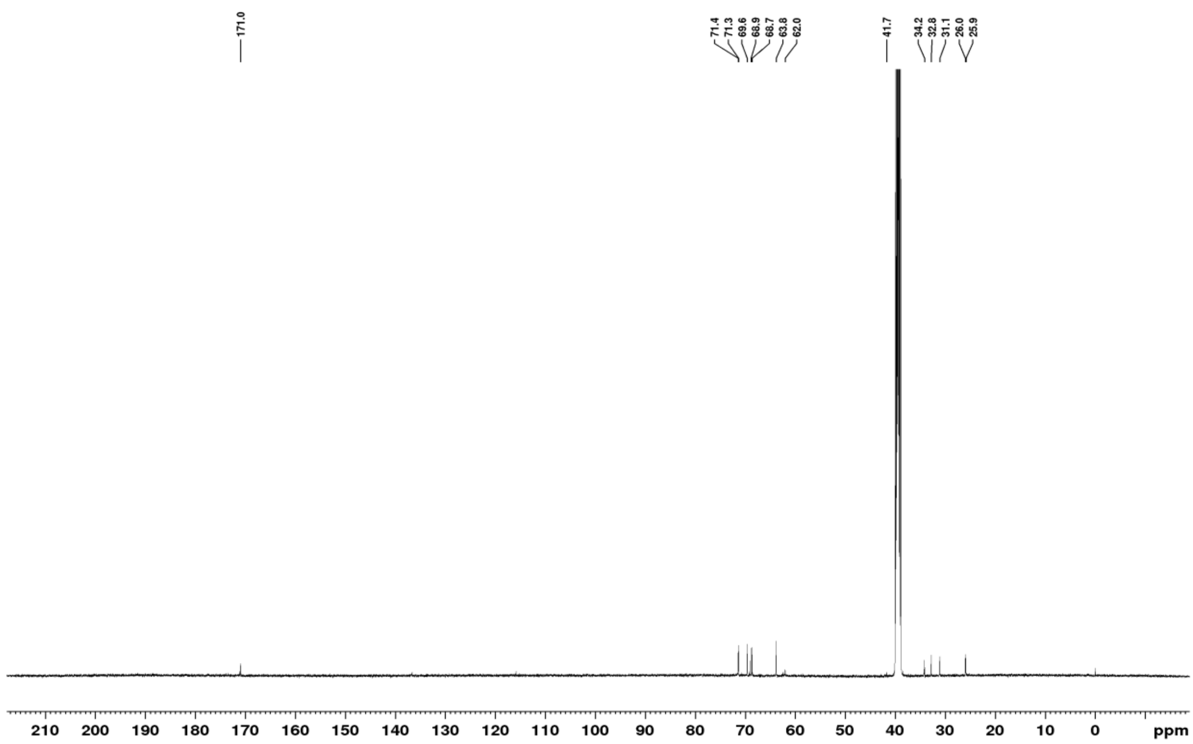

UV-Reactor setup

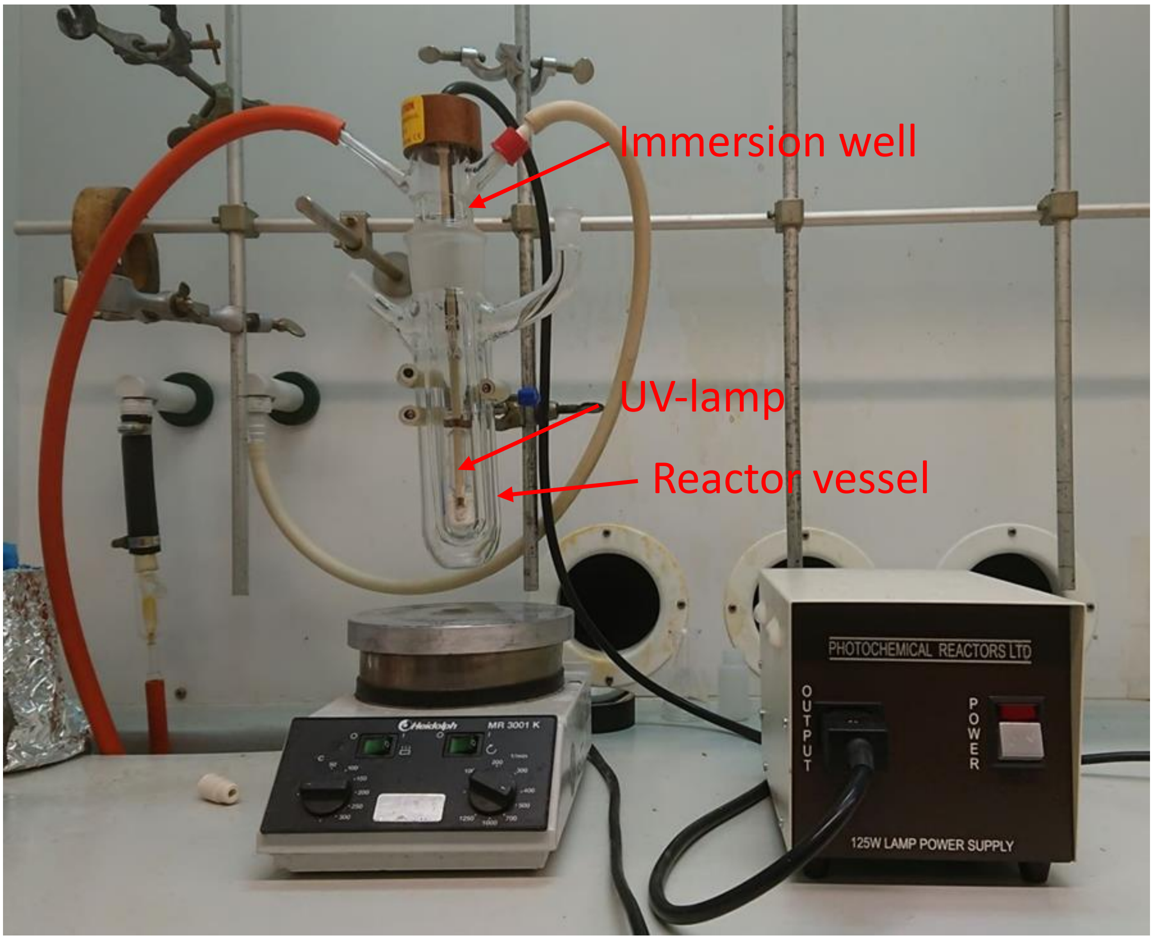

Figure S1. UV-Reactor setup.

Synthesis and NMR spectra of (2*R*,3*R*,4*R*,5*R*,6*S*)-7-(1-(2-hydroxyethyl)-1*H*-1,2,3-triazol-4-yl)heptane-1,2,3,4,5,6-hexaol (**10**)

10 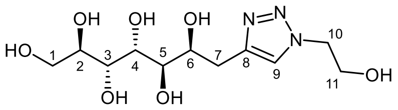

Propargylated D-mannose (20 mg, 0.09 mmol, 1 eq.), azidoethanol (15 mg, 0.18 mmol, 2 eq.), copper(II)sulfate (1.4 mg, 0.009, 0.1 eq.) and sodium ascorbate (3.6 mg, 0.018 mmol, 0.2 eq.) were dissolved in 5 ml distilled water. The solution was heated to 55 °C and stirred at that temperature overnight. The solution was evaporated to near dryness, and 2 ml ethyl acetate was added to precipitate the solids. The solids were washed with 0.5 ml distilled H_2O and were separated from the liquid by centrifugation and decantation, followed by drying under reduced pressure. The reaction yielded 13 mg of white powder (47 %).

^{1}H-NMR spectrum of (2*R*,3*R*,4*R*,5*R*,6*S*)-7-(1-(2-hydroxyethyl)-1*H*-1,2,3-triazol-4-yl)heptane-1,2,3,4,5,6-hexaol (**10**)

10

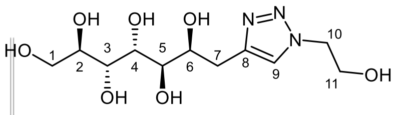

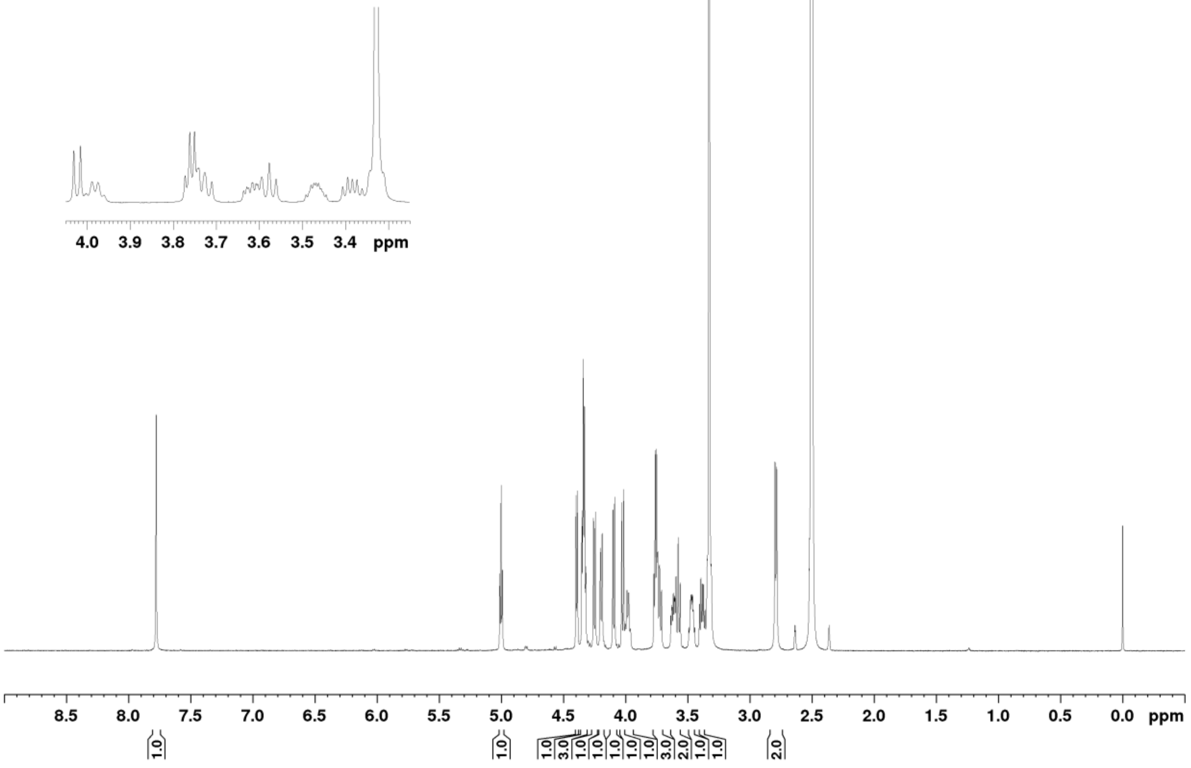

^{13}C-NMR spectrum of (2*R*,3*R*,4*R*,5*R*,6*S*)-7-(1-(2-hydroxyethyl)-1*H*-1,2,3-triazol-4-yl)heptane-1,2,3,4,5,6-hexaol (**10**)

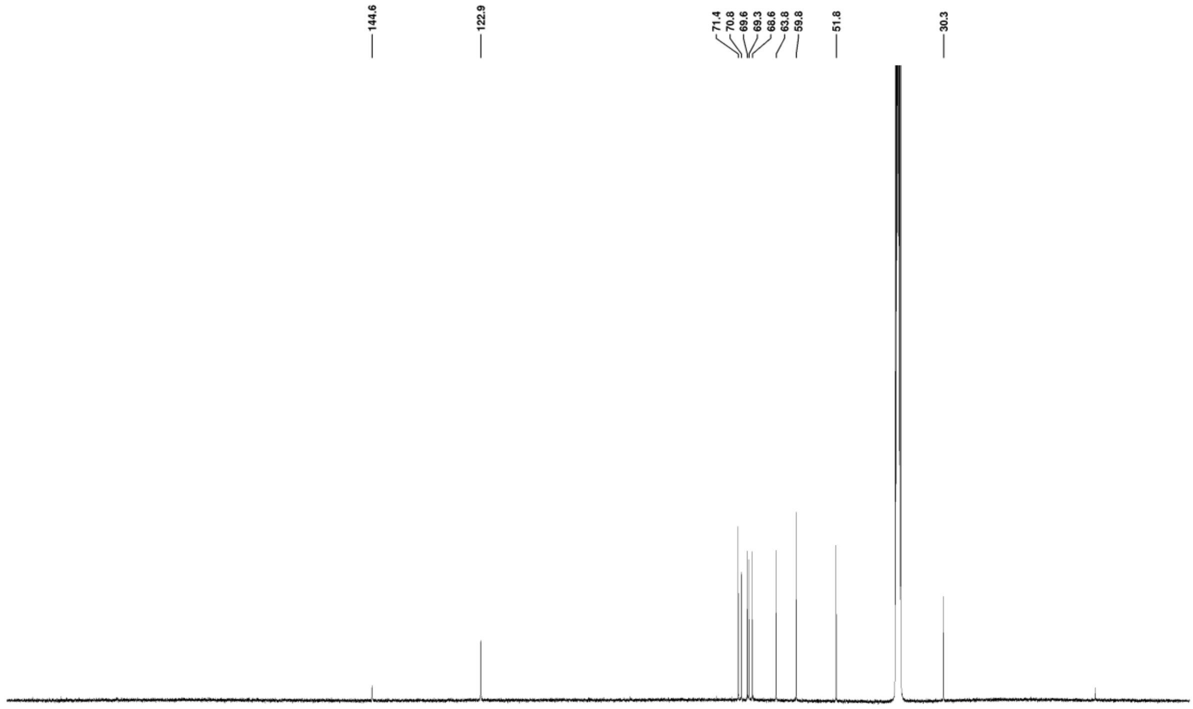

Synthesis and NMR spectra of (2*R*,3*R*,4*R*,5*R*,6*S*)-7-(1-benzyl-1*H*-1,2,3-triazol-4-yl)-heptane-1,2,3,4,5,6-hexaol (11)

11 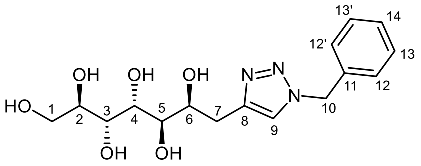

Propargylated D-mannose (20 mg, 0.09 mmol, 1 eq.), azidomethylbenzene (24 mg, 0.18 mmol, 2 eq.), copper(II)sulfate (1.4 mg, 0.009, 0.1 eq.) and sodium ascorbate (3.6 mg, 0.018 mmol, 0.2 eq.) were dissolved in 5 ml H_2O:THF 4:1. The solution was heated to 55 °C and stirred at that temperature overnight. The solution was evaporated to near dryness, and 2 ml toluene was added to remove residual azide. The solids were washed with 0.5 ml distilled H_2O and were separated from the liquid by centrifugation and decantation, followed by drying under reduced pressure. The reaction yielded 18 mg of off-white powder (56 %).

^{1}H-NMR spectrum of (2*R*,3*R*,4*R*,5*R*,6*S*)-7-(1-benzyl-1*H*-1,2,3-triazol-4-yl)heptane-1,2,3,4,5,6-hexaol (**11**)

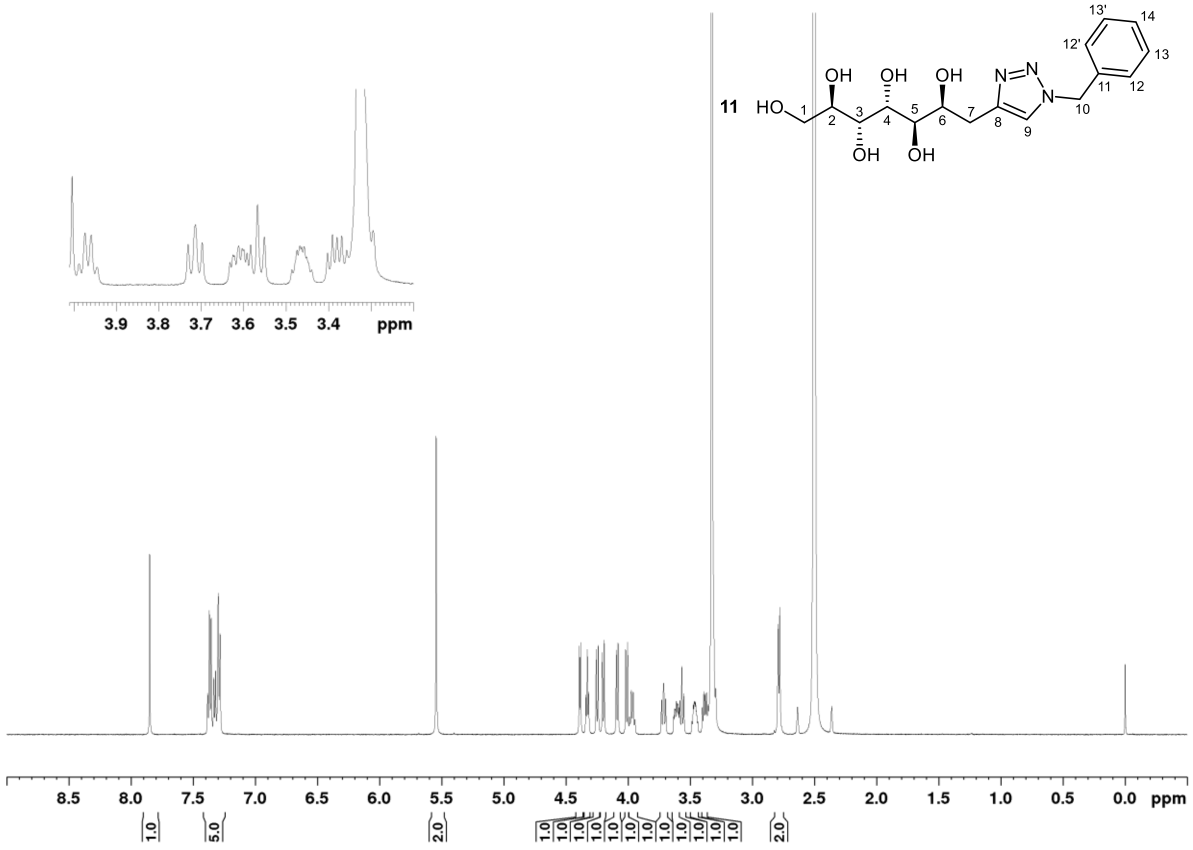

^{13}C-NMR spectrum of (2*R*,3*R*,4*R*,5*R*,6*S*)-7-(1-benzyl-1*H*-1,2,3-triazol-4-yl)heptane-1,2,3,4,5,6-hexaol (**11**)

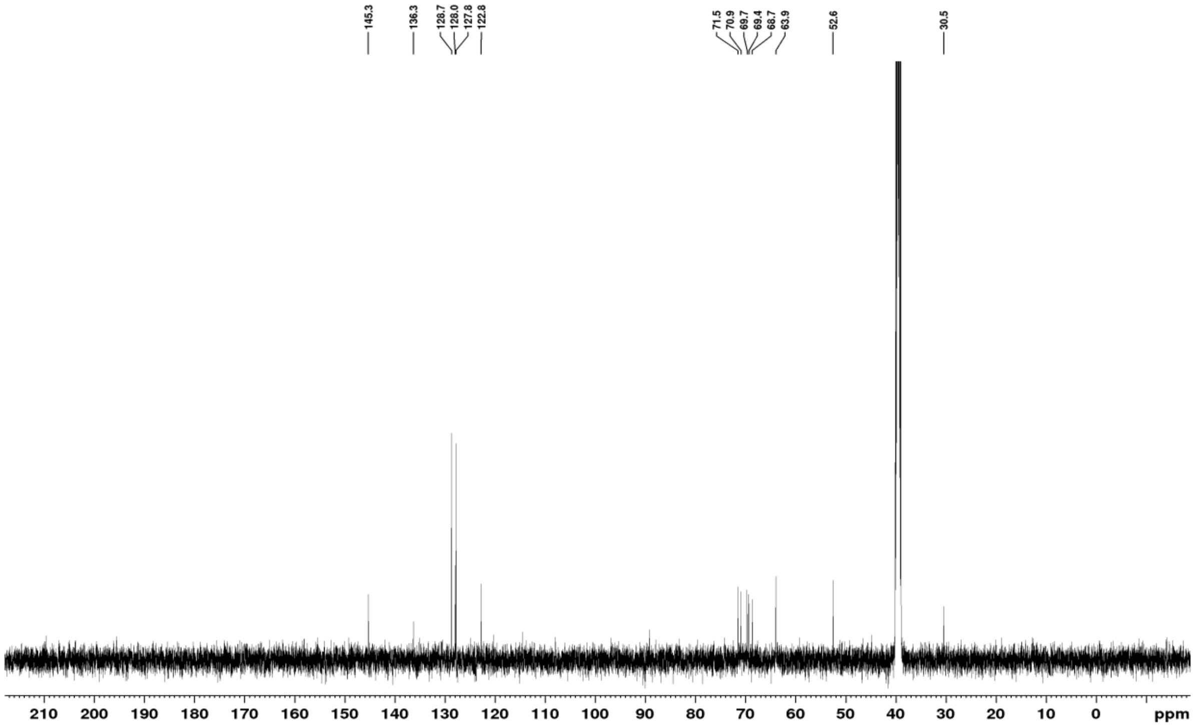

Synthesis and NMR spectra of (2*R*,3*R*,4*R*,5*R*,6*S*)-7-(1-(2-(2-(2-hydroxyethoxy)ethoxy)ethyl)-1*H*-1,2,3-triazol-4-yl)heptane-1,2,3,4,5,6-hexaol (12)

12 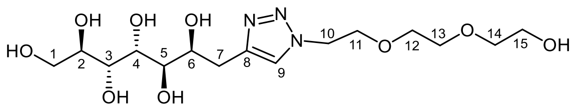

Propargylated D-mannose (20 mg, 0.09 mmol, 1 eq.), 2-[2-(2-azidoethoxy)ethoxy]ethanol (31.5 mg, 0.18 mmol, 2 eq.), copper(II)sulfate (1.4 mg, 0.009, 0.1 eq.) and sodium ascorbate (3.6 mg, 0.018 mmol, 0.2 eq.) were dissolved in 5 ml distilled water. The solution was heated to 55 °C and stirred at that temperature overnight. The solution was evaporated to near dryness, and 2 ml ethyl acetate was added to remove residual azide. The solids were washed with 0.5 ml distilled H_2O and were separated from the liquid by centrifugation and decantation, followed by drying under reduced pressure. The reaction yielded 15 mg of off-white powder (43 %).

^{1}H-NMR spectrum of (2*R*,3*R*,4*R*,5*R*,6*S*)-7-(1-(2-(2-(2-hydroxyethoxy)ethoxy)ethyl)-1*H*-1,2,3-triazol-4-yl)heptane-1,2,3,4,5,6-hexaol (**12**)

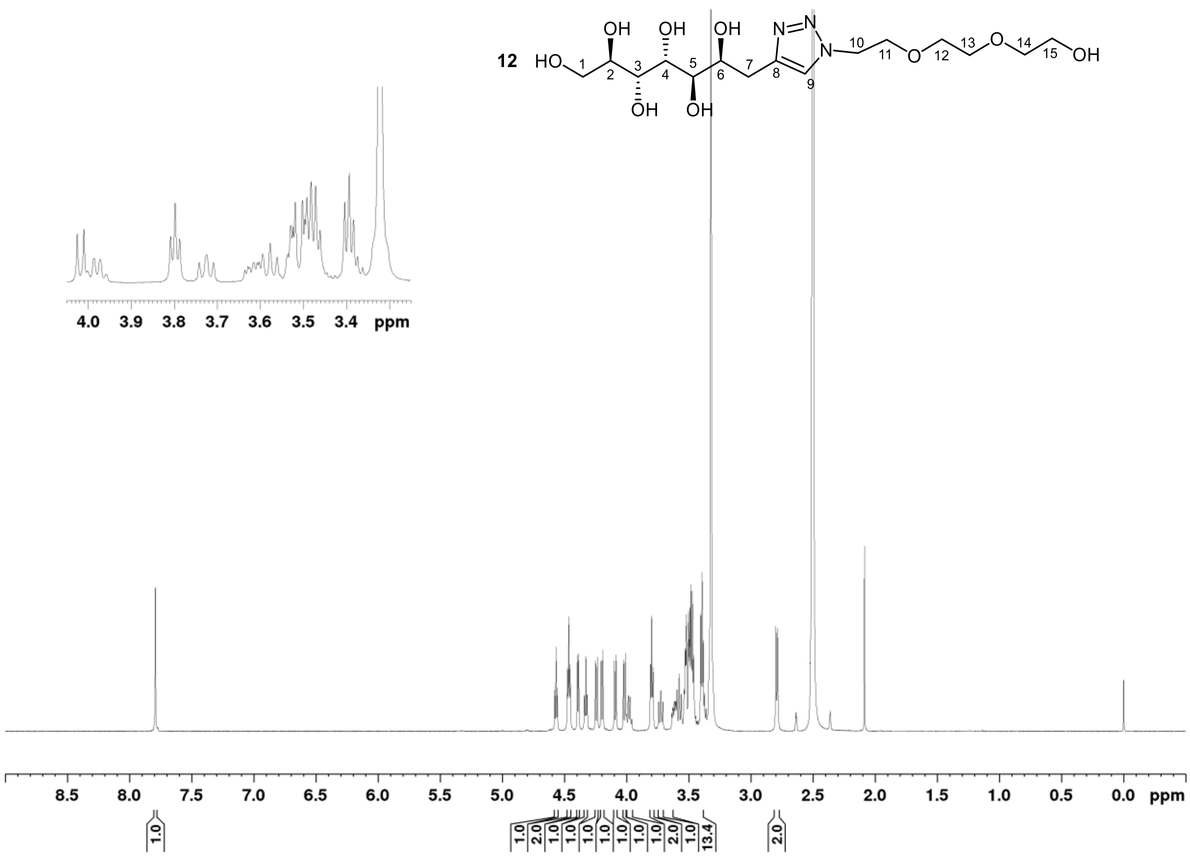

^{13}C-NMR spectrum of (2*R*,3*R*,4*R*,5*R*,6*S*)-7-(1-(2-(2-(2-hydroxyethoxy)ethoxy)ethyl)-1*H*-1,2,3-triazol-4-yl)heptane-1,2,3,4,5,6-hexaol (**12**)

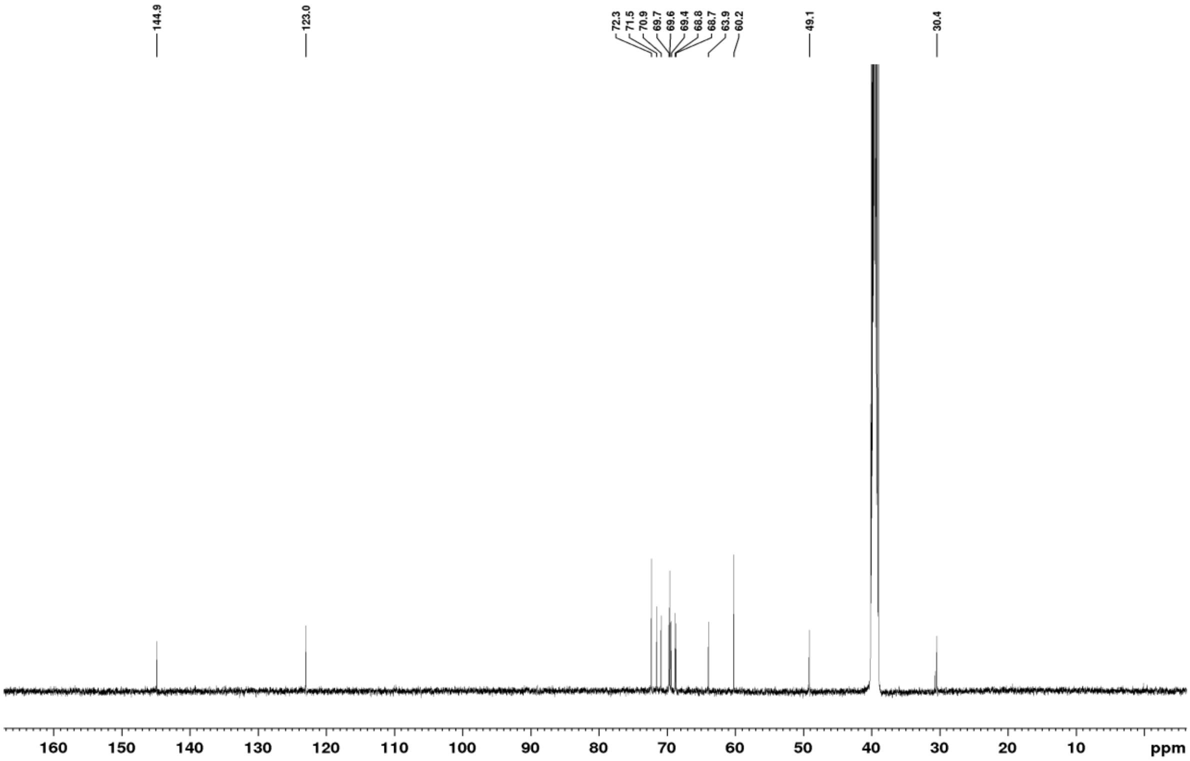

Synthesis and NMR spectra of (2*R*,3*R*,4*R*,5*R*,6*S*)-7-(1-(9*H*-fluoren-9-yl)-1H-1,2,3-triazol-4-yl)heptane-1,2,3,4,5,6-hexaol (**13**)

13 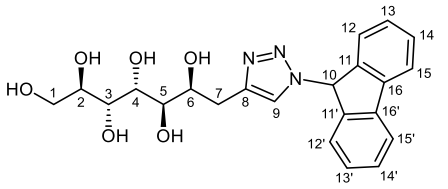

Propargylated D-mannose (20 mg, 0.09 mmol, 1 eq.), 9-azido-9*H*-fluorene (37 mg, 0.18 mmol, 2 eq.), copper(II)sulfate (1.4 mg, 0.009, 0.1 eq.) and sodium ascorbate (3.6 mg, 0.018 mmol, 0.2 eq.) were dissolved in 5 ml DMF:H_2O 4:1. The solution was heated to 55 °C and stirred at that temperature overnight. The solution was evaporated to near dryness, and 2 ml toluene was added to remove residual azide. The solids were washed with 0.5 ml distilled H_2O and were separated from the liquid by centrifugation and decantation, followed by drying under reduced pressure. The reaction yielded 19 mg of off-white powder (49 %).

^{1}H-NMR spectrum of (2*R*,3*R*,4*R*,5*R*,6*S*)-7-(1-(9*H*-fluoren-9-yl)-1H-1,2,3-triazol-4-yl)heptane-1,2,3,4,5,6-hexaol (**13**)

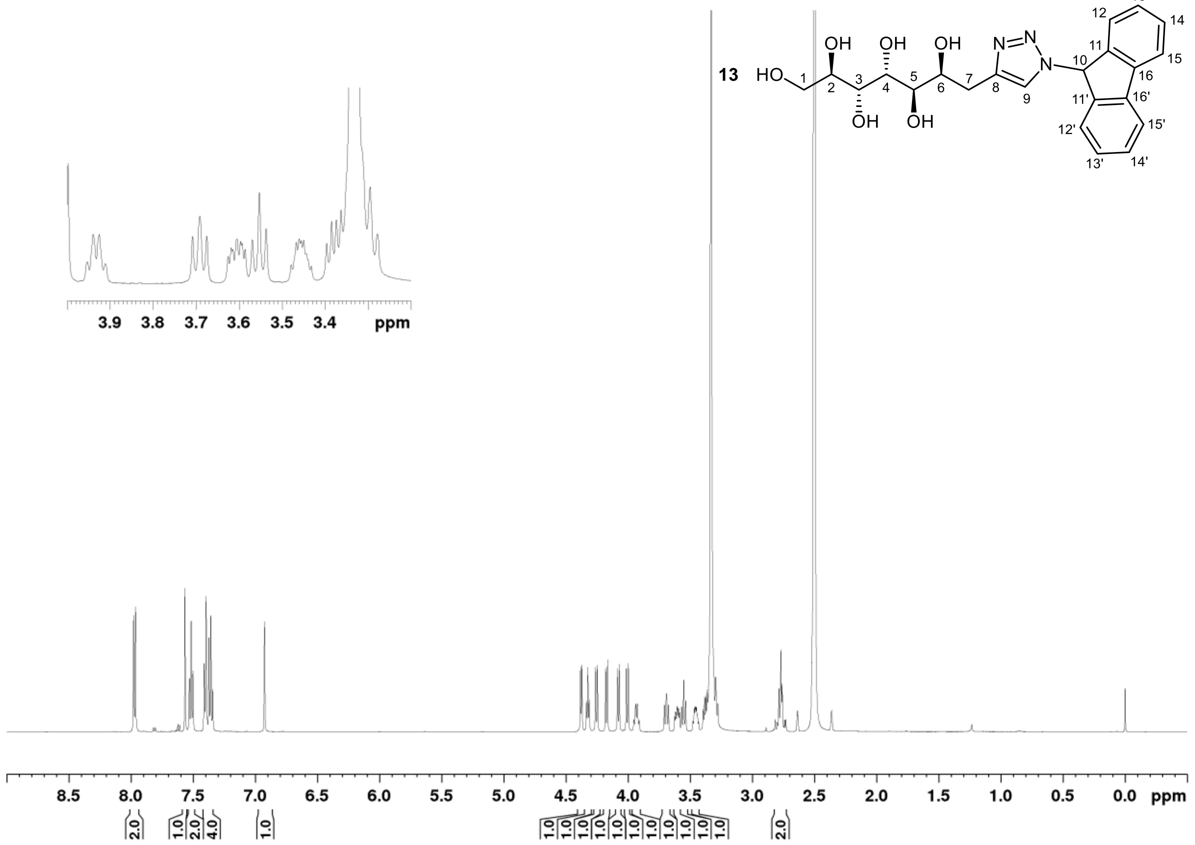

^{13}C-NMR spectrum of (2*R*,3*R*,4*R*,5*R*,6*S*)-7-(1-(9*H*-fluoren-9-yl)-1H-1,2,3-triazol-4-yl)heptane-1,2,3,4,5,6-hexaol (**13**)

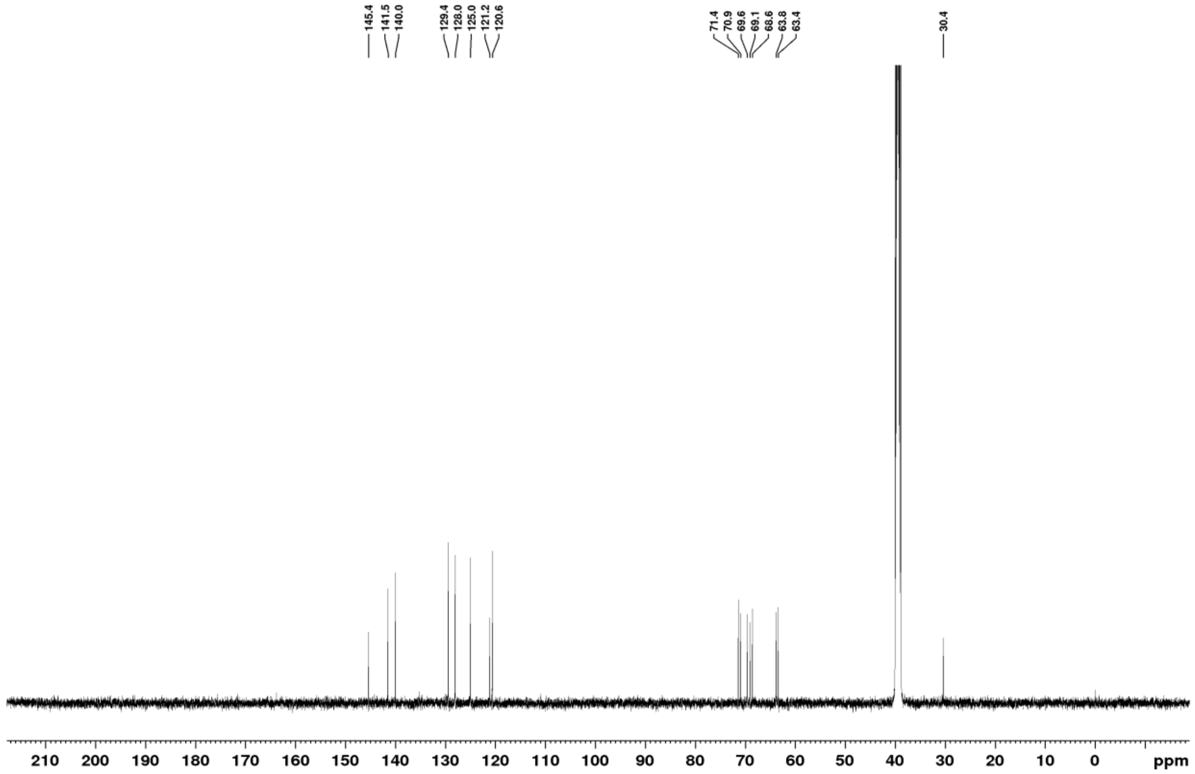

Crystal data of (2*R*,3*R*,4*R*,5*R*,6*S*)-7-(1-benzyl-1*H*-1,2,3-triazol-4-yl)-heptane-1,2,3,4,5,6-hexaol (**11**)

Table S2. Crystal data and refinement parameters of compound **11**.

Identification code	Compound **11**
CCD code	1956436
Empirical formula	$C_{16}H_{23}N_3O_6$
Formula weight	353.37
Crystal system	triclinic
Space group	*P*1
a/Å	4.7849(5)
b/Å	5.3125(5)
c/Å	17.3017(15)
α/°	83.212(8)
β/°	87.407(7)
γ/°	85.031(8)
Volume/Å^3	434.81(7)
Z	1
ρ_{calc} g/cm^3	1.350
μ/mm^{-1}	0.873
F(000)	188.0
Crystal size/mm^3	0.096 × 0.047 × 0.029
2Θ range for data collection/°	5.146 to 153.184
Reflections collected	4839
Independent reflections	2541 [R_{int} = 0.0285, R_σ = 0.0469]
Data/restraints/parameters	2541/3/268
Goodness-of-fit on F^2	1.055
Final R indexes [I>=2σ (I)]	R_1 = 0.0365, wR_2 = 0.0882
Final R indexes [all data]	R_1 = 0.0437, wR_2 = 0.0922
Largest diff. peak/hole / e Å^{-3}	0.23/-0.21

Table S3. Hydrogen bonds in compound **11**.

D	H	A	d(D-H)/Å	d(H-A)/Å	d(D-A)/Å	D-H-A/°
O1	H1	O3[1]	0.82	2.02	2.754(3)	149.4
O2	H2	O1[2]	0.82	1.91	2.717(3)	168.0
O3	H3	O4[3]	0.82	1.88	2.690(3)	172.0
O4	H4	O2[4]	0.82	1.97	2.720(3)	151.3
O5	H5	O6[3]	0.82	1.90	2.713(3)	174.6
O6	H6	N9[1]	0.82	2.08	2.833(4)	152.8
C12	H12	N10[1]	0.93	2.25	3.181(4)	177.1

[1]+X,-1+Y,+Z; [2]-1+X,+Y,+Z; [3] 1+X,+Y,+Z; [4] +X,1+Y,+Z

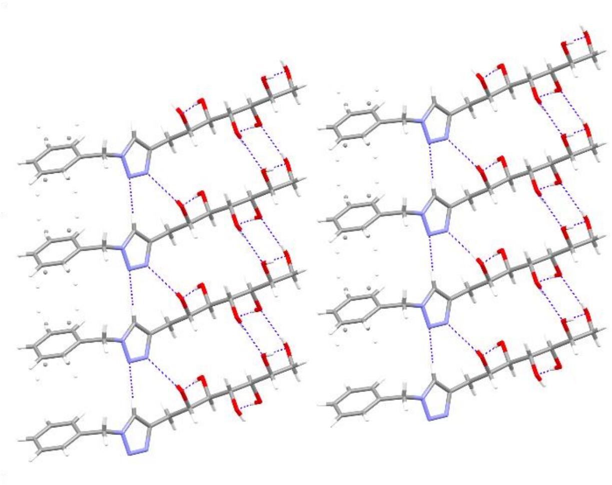

Figure S2. Molecular packing and hydrogen bonding network observed in the structure along *a*-axis in compound **11**. Thermal displacement ellipsoids are displayed at the 50% probability level.
